# Non-invasive ventral cervical magnetoneurography as a proxy of in vivo lipopolysaccharide-induced inflammation

**DOI:** 10.1038/s42003-024-06435-8

**Published:** 2024-07-29

**Authors:** Yifeng Bu, Jamison Burks, Kun Yang, Jacob Prince, Amir Borna, Christopher L. Coe, Alan Simmons, Xin M. Tu, Dewleen Baker, Donald Kimball, Ramesh Rao, Vishal Shah, Mingxiong Huang, Peter Schwindt, Todd P. Coleman, Imanuel Lerman

**Affiliations:** 1 Department of Electrical and Computer Engineering, University of California San Diego, La Jolla, CA 92093 USA; 2 Department of Bioengineering, University of California San Diego, La Jolla, CA 92093 USA; 3 Division of Biostatistics and Bioinformatics, University of California San Diego, La Jolla, CA 92093 USA; 4 Quantum Information Sciences, Sandia National Laboratories, Albuquerque, NM 87123 USA; 5 Department of Psychology, University of Wisconsin-Madison, Madison, WI 53706 USA; 6 Center for Stress and Mental Health (CESAMH) VA San Diego, La Jolla, CA 92093 USA; 7 Department of Psychiatry, University of California San Diego, La Jolla, CA 92093 USA; 8 Quspin Laboratory Head Quarters, Boulder, CO 80305 USA; 9 Department of Radiology, University of California San Diego, La Jolla, CA 92093 USA; 10 Department of Bioengineering, Stanford University, Stanford, CA 94305 USA; 11 InflammaSense Incorporated Head Quarters, La Jolla, CA 92093 USA; 12 Department of Anesthesiology, University of California San Diego, La Jolla, CA 92093 USA

**Keywords:** Autonomic nervous system, Neuroimmunology

## Abstract

Maintenance of autonomic homeostasis is continuously calibrated by sensory fibers of the vagus nerve and sympathetic chain that convey compound action potentials (CAPs) to the central nervous system. Lipopolysaccharide (LPS) intravenous challenge reliably elicits a robust inflammatory response that can resemble systemic inflammation and acute endotoxemia. Here, we administered LPS intravenously in nine healthy subjects while recording ventral cervical magnetoneurography (vcMNG)-derived CAPs at the rostral Right Nodose Ganglion (RNG) and the caudal Right Carotid Artery (RCA) with optically pumped magnetometers (OPM). We observed vcMNG RNG and RCA neural firing rates that tracked changes in TNF-α levels in the systemic circulation. Further, endotype subgroups based on high and low IL-6 responders segregate RNG CAP frequency (at 30-120 min) and based on high and low IL-10 response discriminate RCA CAP frequency (at 0-30 min). These vcMNG tools may enhance understanding and management of the neuroimmune axis that can guide personalized treatment based on an individual’s distinct endophenotype.

## Abstract (web summary)

Ventral cervical magnetoneurography responses after LPS administration temporally aligned with the changes in TNF-α levels, making it a potential sensitive bioindicator of the neuroimmune axis for clinical use.

## Introduction

Autonomic homeostasis is continuously calibrated by sensory fibers of the vagus nerve and sympathetic chain that communicate in a reciprocal manner with the central nervous system via compound action potentials (CAPs)^1–3^. Afferent autonomic sensory neurons are responsive to blood pressure, mechanical stretch, temperature, metabolites, and pH, and they integrate and transmit cumulative action potentials to the spinal cord and brainstem^4–8^. Significant physiological autonomic nervous system (ANS) change consistently occurs in response to the innate immune system activation due to bacterial infection and is mimicked by injection of lipopolysaccharide (LPS), which resembles acute endotoxemia^9,10^. Core to the LPS-induced host response, the activation of peripheral immune cells results in increased cytokine secretion into the systemic circulation, which stimulates afferent sensory neuronal fibers of both the vagus and sympathetic chains; activation of these afferent neuronal sensory fibers can be detected via CAPS^11–13^. To date, these afferent sensory neuronal fiber recordings required invasive penetrating or cuff electrodes, while non-invasive clinically capable recording methods have as of yet, been employed to monitor these autonomic neural components (i.e.,vagus nerve, jugular and nodose ganglion, carotid sinus nerve).

Our group recently developed a wearable ventral cervical electroneurographic system capable of recording neural action potentials emanating from the ANS^14^. Leveraging parallel advances in signal processing methods for cortical^15–18^ and cuff electrode recordings^19–21^, we developed a surface electrode array that recorded ventral cervical autonomic neurography (vcANG) across several different autonomic challenges, (i.e., cold pressor test and a timed deep respiratory challenge)^14^. Consistent responses were detected across a number of autonomic challenges, including vcANG and cardiac autonomic measures (CAM). The amplitude and frequency of vcANG identified distinct subgroups, who were classified into several endo-specific phenotypes^14^. Ventral cervical neuronal tracts (including vagus nerve and sympathetic chain) can also quantify the autonomic physiological response to an LPS challenge in a dose-dependent manner^19,22,23^. Further, a growing body of literature has consistently documented that CAM can reflect a significant decrease in vagal activity as measured by heart rate variability (HRV) in healthy subjects challenged with intravenous LPS^23–26^. In the clinical disease, chronic rheumatoid arthritis (RA), patients evince a larger reduction in vagal HRV indices when compared to less severe RA^27–29^. These observations implicate baseline aspects of reflex autonomic tone as a continuous regulator and potential bioindicator of peripheral inflammatory activity. Other disease states, such as Post Traumatic Stress Disorder (PTSD) are also known to present with low vagal HRV indices^30–32^ and exhibit hyperinflammation, both at rest^33^ and in response to the arousing task of reading a script describing traumatic events^34–42^. Collectively, the evidence indicates that there is a previously undiscovered relationship between dysregulated CAM and peripheral inflammation, which can also be leveraged to assess disease severity.

Beyond the specific aim of demonstrating that conventional CAM bioindicators of ANS change in response to intravenous administration of LPS, our group recently found that non-invasive OPMs (bandwidth increased to 500 Hz) are capable of tracking evoked peripheral nerve action potentials when positioned over peripheral nerve targets (i.e., the median nerve of the wrist, forearm, and axilla), termed as “magnetoneurography” (MNG)^43^. Building on this work, OPMs were positioned ventrally over autonomic neural targets of interest: (1) rostral sensors were placed over the ventral cervical area overlying the jugular and nodose ganglion and (2) caudal sensors were placed over the ventral cervical area overlying the carotid sinus and vagus nerve. Recordings at each site were carried out during a human intravenous LPS injection challenge. The aim was to enable ventral cervical MNG (vcMNG) to track ANS changes in response to an LPS challenge in humans.

It is well established that an intravenously injected LPS will significantly increase peripheral blood cytokine levels (i.e., over the 7-hr protocol used in the current work)^44,45^. Both LPS and the administration of inflammatory cytokines will also elicit increases in afferent vagal^11–13^ and afferent sympathetic nerve firing frequency^13^. In fact, recent work has shown that it is possible to isolate and decode cytokine-specific vagus neural firing signals as expressed in CAPs format^19^. This phenomenon was observed for IL-1β and TNF-α via cuff electrode recordings of ventral cervical vagal activity^19–21,46–48^. TNF-α concentration was similarly correlated with carotid sinus nerve firing activity (a branch of the sympathetic chain located in the ventral cervical carotid sheath), further highlighting that ventral cervical neurons are responsive to an inflammatory challenge^13^. Furthermore, preclinical, inflammatory challenge models (including LPS, IL-1, and IL-1ra) have shown repeatedly that the neural response firing of the vagus and sympathetic tracts (i.e., carotid sinus, spleen, renal nerve) change in a dose-response manner^13,19,21,46,47,49–52^ (for review see Griton et al. ^53^). Although the specific neuroanatomical pathways are still debated^1^, the general consensus indicates that afferent ventral cervical neural signaling (i.e., sensory vagus and sympathetic chain nerve CAPs) result in an efferent arc signaling either directly or indirectly through changes in splenic sympathetic neural activity that regulates immune cell signaling at the spleen^11,51^, bone marrow, lymph nodes^54,55^ and thymus^56,57^.

Our primary aim was to noninvasively characterize the human ventral cervical neuronal response to a standard LPS challenge. vcMNG allowed for real-time recording of cytokine-induced ventral cervical neural responses, which were indexed by changes in firing frequency capable of capturing neuroimmune axis reactivity. Collectively, vcMNG is poised to provide benchmark metrics of host variation in neuro-inflammatory responses that are pathogen-agnostic. These changes in regulatory and adaptive physiology can be stratified to identify the likelihood of excessive or ineffective inflammatory immune responses in the clinical setting.

## Results

**Fig. 1:**
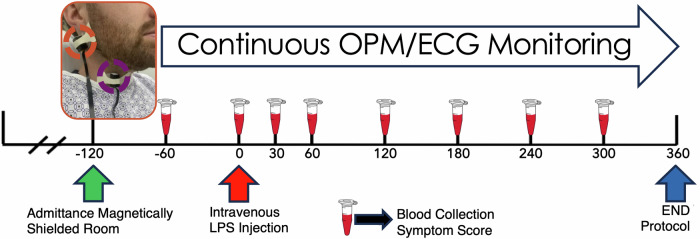
Human intravenous Lipopolysaccharide (LPS) injection protocol. Participants admitted to the magnetically shielded underwent physical screening and exam clearance. Optically Pumped Magnetometers (OPMs) were positioned over the Right Nodose Ganglion (RNG) (orange dashed circle) and Right Carotid Artery (RCA) (purple dashed circle) while standard Electrocardiocagraphy (ECG) was recorded with conventional surface electrodes. Blood samples and symptom severity scores were collected at –60, 0, 30, 60, 120, 180, 240 and 300 min. 3 ng/kg LPS was administered at 0-minute time point. Continuous OPM and ECG recordings were monitored from -60 to 360-minute time points.

Twelve physically healthy male participants were recruited for this study with a median age of 20 yr (18–30 yr) and a median weight of 71.5 kg (52–118 kg, BMI: 22.86 ± 4.95) (Supplementary Table 1). On the test day, subjects were screened with a physical history and exam clearance, which excluded subjects with prior cardiac illness or a history of syncope. The screening session was followed by the insertion of two large-bore intravenous catheters placed within the antecubital fossa. One subject was excluded due to exhibiting syncopal signs and symptoms upon intravenous catheter insertion. Throughout the protocol, electrocardiography (ECG) and OPM ventral cervical MNG (vcMNG) were recorded at two separate sites, which were positioned over the rostral Right Nodose Ganglion (RNG) and caudal Right Carotid Artery (RCA) (Fig. 1). Unilateral, right-sided vcMNG was recorded as right-sided human vagus cadaveric studies demonstrated a greater number of vagus fibers when compared to the left side^58^. Two subjects experienced nausea and emesis, after which the magnetically shielded room (MSR) was opened for care prior to halting data acquisition of magnetic field signals; in these two subjects, OPM saturation precluded further data acquisition. In summary, integrated recordings from nine subjects were included in the final analyses.

### Intravenous LPS injection elicits significant changes in magnetoneurography (MNG) activity

**Fig. 2:**
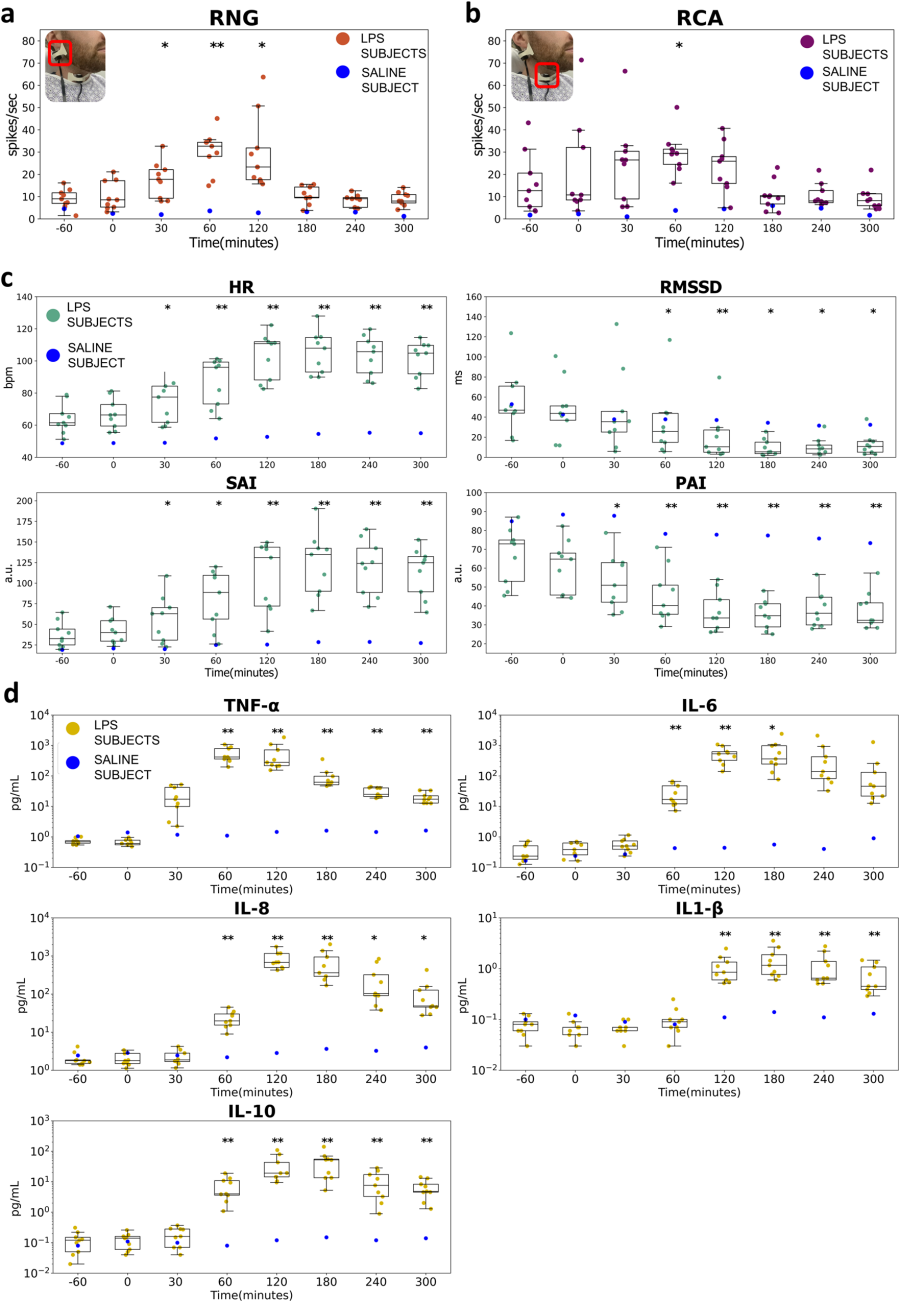
Alterations in Magnetoneurography (MNG) over the Right Nodose Ganglion (RNG), Right Carotid Artery (RCA), cardiac autonomic measures (CAM), and cytokine levels pre-to-post LPS challenge. Alterations in Magnetoneurography (MNG) of optically pumped magnetometer sensors placed over the RNG (**a**) and RCA (**b**). **c** Alterations in cardiac autonomic measures (CAM) pre-to-post LPS injection (HR: heart rate, RMSSD: root mean square of successive differences, SAI: sympathetic activity index, PAI: parasympathetic activity index). **d** Changes in cytokine concentrations TNF-α, IL-6, IL-8, IL-1β, IL-10 levels pre-to-post LPS injection. The actual measurement from each subject at each time point is overlaid on the boxplot. The results from a healthy control are presented as additional blue dots in each subplot. The statistical comparisons and significance of each metric post-LPS to baseline levels are marked with asterisk symbols (*p < 0.05, **p < 0.01). Changes from the baseline of continuous recordings (i.e., RNG, RCA, CAM) were calculated through generalized estimating equations (GEE) for each session (See “Methods” for more details). Discrete measurements of cytokine responses were first evaluated with GEE to confirm the overall main effect of time, and then two-way paired t-tests were performed for baseline pre-to-post-LPS (See “Methods” for more details). Boxplot centerline: median; box limits: Q1 and Q3 quantile.

Raw neural recordings include structured noise from cardiac, respiratory, and tremor activity. Therefore, a 50–500 Hz bandpass filter was applied to separate the noise signal from the raw data. As described in the “Methods” section, a constant thresholding technique was used to identify candidate neural spikes. An example spike waveform is shown in Supplementary Fig. 1. The key feature extracted from the processed MNG data was the firing rate based on the thresholded spikes, where the rate is calculated by counting the number of spikes per second. Aggregate RNG firing rates revealed significant change with respect to baseline from 30-to-120 min (Fig. 2a), while aggregate RCA firing rate showed significance only at the 60-min time point (Fig. 2b). Exemplary recorded data at RNG and RCA after signal processing and spike detection from an LPS subject was shown in Supplementary Fig. 2. As a comparison, vcMNG recorded at the identical location (RNG and RCA) from a healthy control following intravenous normal saline injection only is presented as blue dots in Fig. 2 and in Supplementary Fig. 3.

### Intravenous LPS injection elicits significant changes in heart rate and HRV

CAM of heart rate and three different HRV metrics showed that there were significant changes in activity post-LPS injection (Fig. 2c). Heart rate and cardiac-related HRV underwent sustained changes throughout the trial. The root mean square of successive differences (RMSSD) was chosen as a time-domain indicator of HRV which is believed to optimally reflect parasympathetic activity^59^; it is similarly sensitive for the prediction of septic shock^60,61^. In contrast, traditional frequency domain measures (i.e., low-frequency power and high-frequency power) have been questioned because they cannot efficiently delineate and separate the sympathetic and parasympathetic nervous system activity^62–64^. Instead, the Sympathetic Activity Index (SAI) and Parasympathetic Activity Index (PAI) were selected as two alternative heart beat-derived autonomic measures, which are evaluated through the expansion of Laguerre and Volterra functions (see “Methods”)^65,66^. Both SAI and PAI have outperformed traditional frequency-domain measurements in tracking expected variation in instantaneous sympathetic and parasympathetic activity in classical autonomic challenges^65,66^. Heart rate, SAI, and PAI metrics increased significantly at 30 min, while RMSSD of the RR intervals significantly increased later at the 60-min time point (*p* < 0.05) (Fig. 2c).

### Cytokine levels increase significantly in response to intravenous LPS

LPS elicited a robust increase in all cytokines in peripheral blood (Fig. 2d). The time-to-increase and time-to-peak analyses provided a representative temporal data structure of the response of the 5 cytokines (i.e., TNF-α, IL-6, IL-8, IL-1β, IL-10). These cytokines are known to increase in clinical diseases such as sepsis^26^ and high TNF-α concentrations are predictive of morbidity and mortality^67^. To verify the changes in cytokine levels pre-to-post injection of LPS, an omnibus test was run. It accounted for potential unequal variance by deploying generalized estimating equations (GEE), and it controlled for multiple comparisons. This analysis affirmed a main effect of time on all cytokines (p < 0.001). Significant changes in cytokine levels from baseline (-60 min) to post-LPS injection (measured at 0-, 30-, 60-, 120-, 180-, 240-, and 300-min) were identified by the paired t-tests (Fig. 2d). Circulating levels of TNF-α were significantly increased at the 30-min time point (*p* < 0.01), while significant increments in IL-6 and IL-8, were evident at the 60-min time point (*p* < 0.01). IL-1β showed the latest significant change at 120 min (*p* < 0.01). TNF-α, IL-8, and IL-1β remained significantly elevated at the final blood draw (300 min post-injection; *p* < 0.05), whereas IL-6 was significantly different from baseline only until 180 min (*p* < 0.05). The regulatory and anti-inflammatory cytokine, IL-10, increased at the 60-min time point and remained elevated throughout the protocol (*p* < 0.01). Mean peak concentrations for TNF-α, IL-6, IL-8, IL1-β, and IL-10 were 719.41 pg/mL, 808.90 pg/ml, 924.65 pg/mL, 1.61 pg/mL, and 53.12 pg/mL respectively. The levels of other cytokines, including IL-4, IL-12p70, IL-13, and IFN-ϒ, pre-to-post LPS injection are illustrated in Supplementary Fig. 4. Physiological neural firing, heart rate, CAM, and cytokine changes in a single healthy control who received only intravenous saline are shown as additional blue colored dots in Fig. 2 and Supplementary Fig. 4a. No large or consistent changes in neurological, physiological, or cytokines were observed in the healthy control subject who received only intravenous normal saline (Fig. 2 and Supplementary Fig. 4a).

### Correlation among neurological, physiological, and biological features

**Fig. 3:**
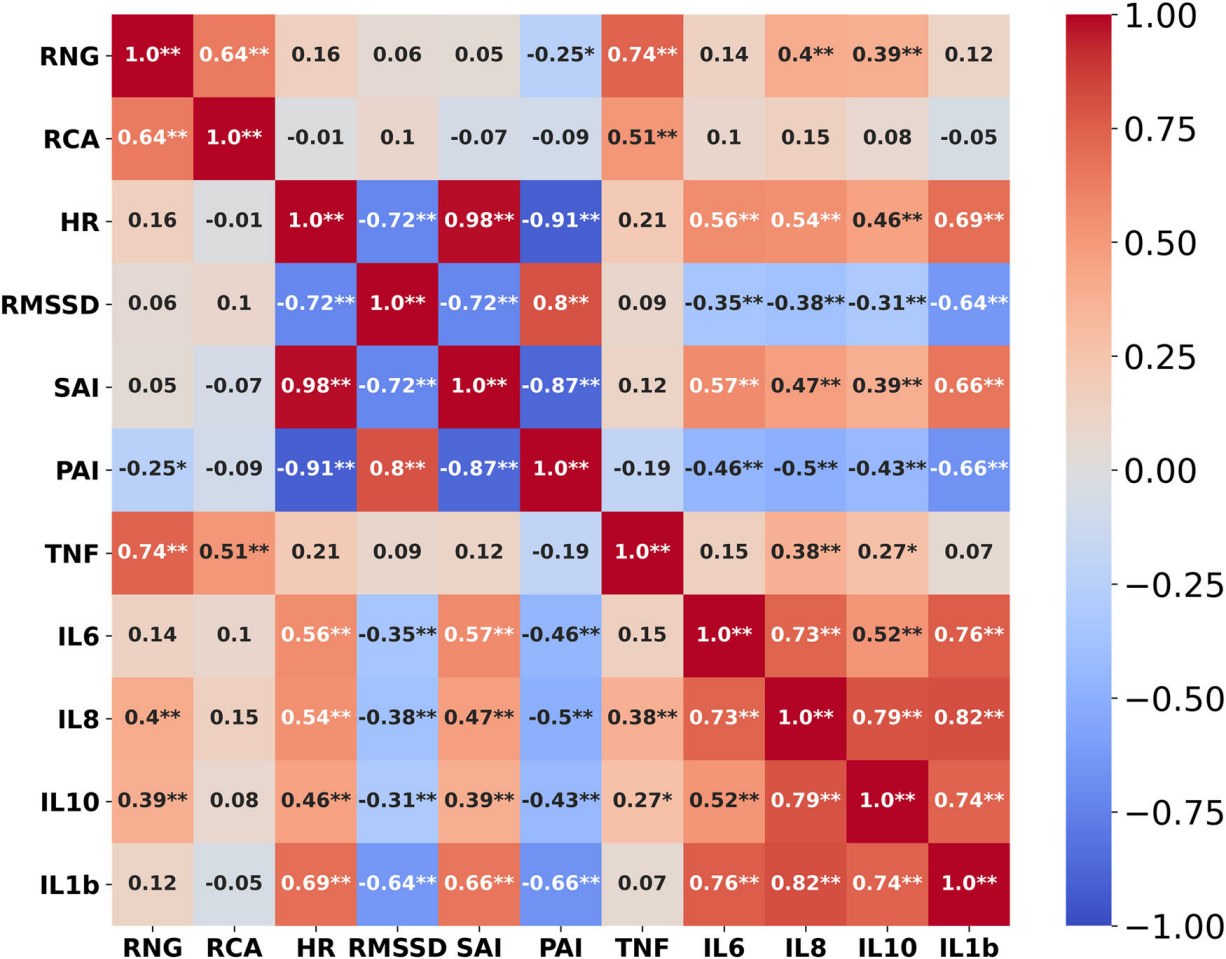
Repeated measure correlation (rmcorr). The heat map shows pairwise rmcorr and their associated significance levels among neurological (i.e., RNG, and RCA neural firing), physiological (i.e., CAMs), and biological (i.e., cytokines) features for all subjects (* *p* < 0.01, ** *p* < 0.001).

Pairwise correlations among neural sensors (i.e., RNG and RCA neural firing), physiological (i.e., CAMs), and biological (i.e., cytokines) features were analyzed using the repeated measure correlation (rmcorr, see “Methods”). Rmcorr estimates a common regression slope shared among multiple individuals and its associated significance while not violating the assumption of independent observation. Improved statistical power can be obtained with rmcorr because it does not average or aggregate the intra-individual comparisons^68^. In Fig. 3, the pairwise rmcorr and its associated significance level are presented as a heatmap. Three findings emerged from this analysis. First, neurological features and physiological features showed high and significant correlations within each category, while the correlation between these two categories was weak. Second, the correlation between TNF-α and the other four cytokines (i.e., IL-6, IL-8, IL-10, IL1-β) was weak compared to the inter-relationships among the other four cytokines. Third, TNF-α was more clearly correlated with the neurological features while no correlations were observed with any physiological features. In contrast, the other four cytokines were more significantly associated with physiological features.

### High- and low-responders are identified by cytokine profiles

**Fig. 4:**
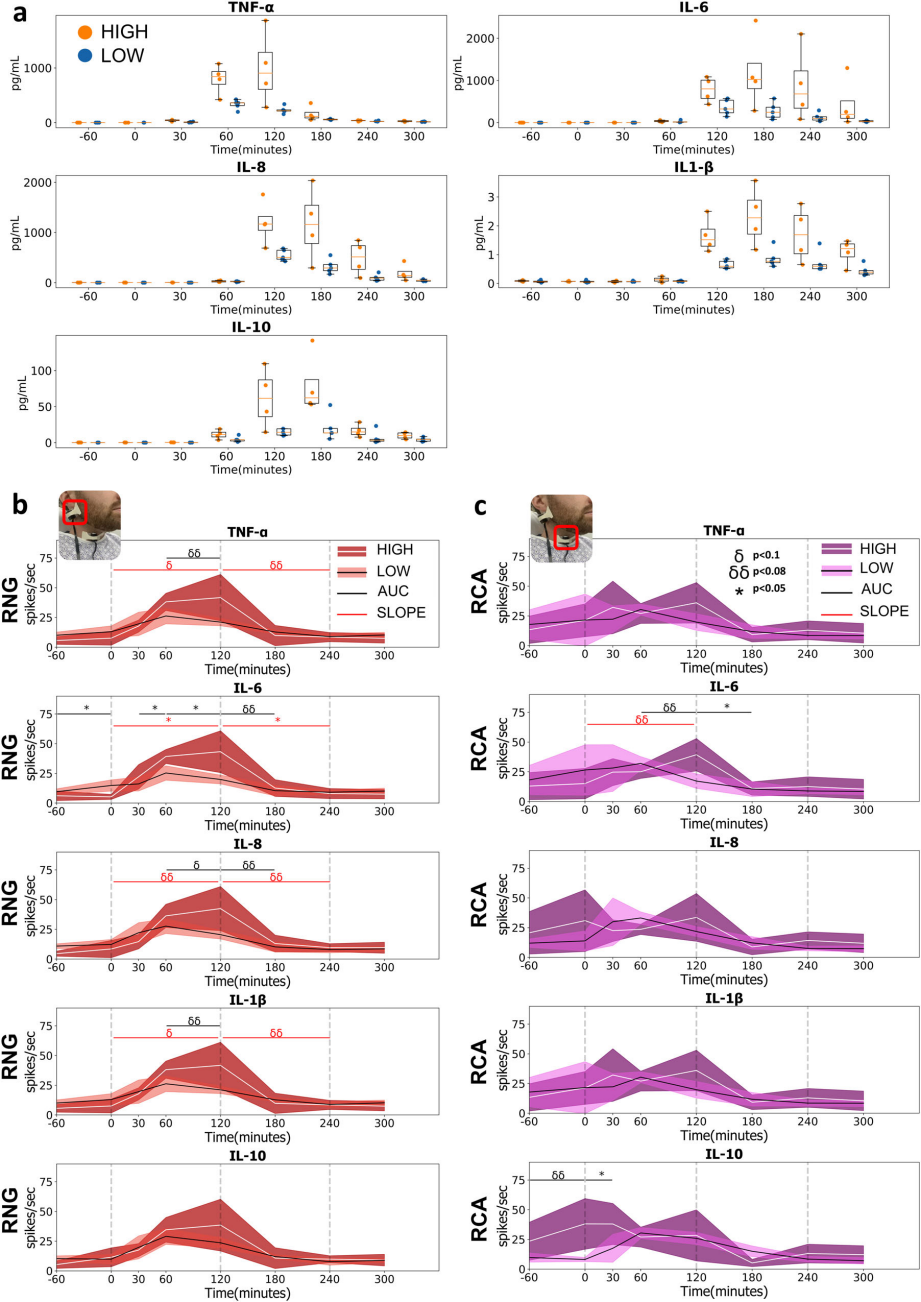
Variation in cytokine release and MNG neural activities across time for the high and low responder subgroups. **a** Individual variation in cytokine release for the high and low responder subgroups. Using the median of the peak cytokine concentration for all participants as a threshold, individuals were categorized into high and low-responder subgroups for each cytokine. Boxplot centerline: median; box limits: Q1 and Q3 quantile. Change in MNG activity at the RNG (**b**) and RCA (**c**) pre-to-post injection of LPS for high and low responder subgroups for each cytokine was recorded, and the shaded areas represented the 95% confidence interval. Two statistical comparisons were conducted. (1) For each session (between each blood draw), the cumulative change (RNG or RCA firing frequency) area under the curve (AUC) was compared between high and low responders across the 7 phase intervals (-60-to-0 min, 0-to-30 min, 30-to-60 min, 60-to-120 min, 120-to-180 min, 180-to-240 min, 240-to-300 min). Significant AUC between subgroup differences over 7 measured phase intervals was marked by horizontal black lines (*=*p* < 0.05, δ = *p* < 0.08, δδ = *p* < 0.1). (2) Changes in MNG activity over the 0–120 min and 120–240 min time intervals were designated as increasing and decreasing CAP slope. The slope of CAP firing frequency over these periods (0–120 and 120–240 min) compared between high and low responder groups; significant comparisons were demarcated by the horizontal red line (*=*p* < 0.05, δ = *p* < 0.08, δδ = *p* < 0.1).

**Fig. 5:**
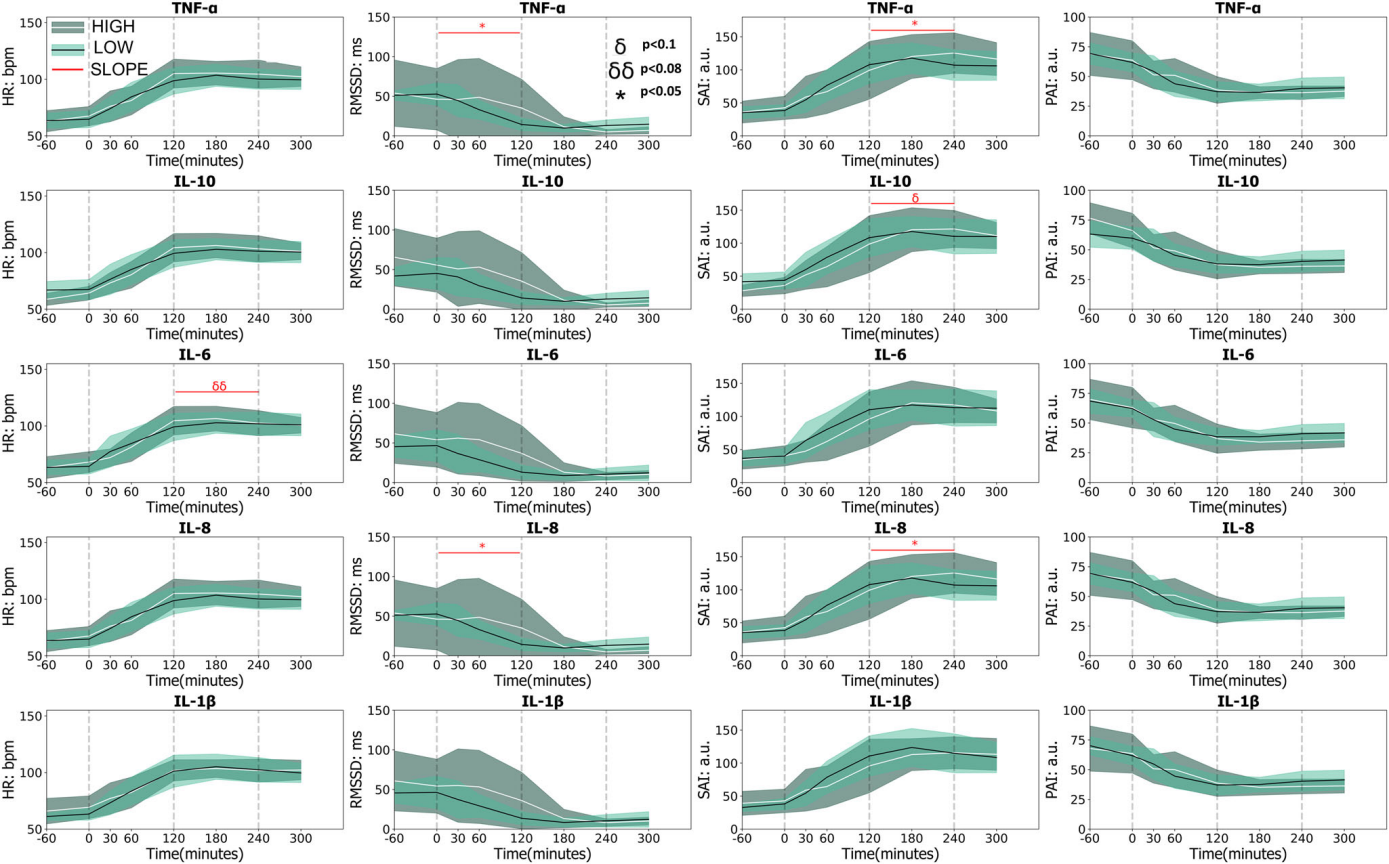
Variation in CAM across time for high and low responder subgroups. High and low responders were based on TNF-α, IL-6, IL-8, IL-1β, and IL-10, and revealed AUC and slope difference in cardiac autonomic measures (CAM). The graphs illustrate temporal changes in Heart Rate (HR), Root Mean Square of Successive RR intervals (RMSSD), Sympathetic Activity Index (SAI), and Parasympathetic Activity Index (PAI) pre-to-post injection of LPS. The 95% confidence intervals are portrayed by the shaded areas. For each session (between each blood draw), the cumulative change area under the curve (AUC) was compared between high and low responders. Subgroup (high and low) did not demonstrate any significant difference in AUC for any CAM. The slope of CAM over periods 0-120 and 120-240 min were compared between high and low responder subgroups; significant slope differences during these two periods were demarcated by horizontal red lines and red symbols (*=p < .05, δ = p < .08, δδ = p < .1).

Participants were categorized into high- and low-responder subgroups using the median of peak concentration of each cytokine (i.e., TNF-α, IL-6, IL-8, IL-1β, IL-10) (Fig. 4a). Subgroup vcMNG activity at the RNG and RCA sites (aggregate CAP firing frequency across the LPS challenge protocol) was compared between high and low responders for each cytokine (Fig. 4b, c). For the RNG site (aggregate CAP firing frequency AUC), subgroups segregated: (1) significantly for IL-6 over the 30-to-120 min interval, (2) at the trend level for TNF-α and IL-1β over the 60-to-120 min intervals (*p* < 0.08), and (3) at a trend level for IL-8 over the 60-to-180 min interval (Fig. 4b). Of note, no RNG site (aggregate CAP firing frequency AUC) subgroup differences were observed for IL-10 (Fig. 4b). Similar to the RNG site, the RCA site (aggregate CAP firing frequency AUC) subgroups segregated: (1) significantly for IL6 between the 120-to-180 min interval, and (2) at the trend or significant level for IL-10 over the -60-to-30 min interval (Fig. 4c). In stark contrast, subgroup designation did not segregate significant AUC change in heart rate or other HRV metrics in any phase of the session for all cytokines (Fig. 5).

Slopes of aggregate CAP firing frequency changes over two time periods (0-to-120 min and 120-to-240 min) were different in the two subgroups: (1) there was a significant or trend level difference in both periods for TNF-α, IL-6, IL-8, and IL-1β at the RNG sensor site (Fig. 4b). In contrast, at the RCA sensor site, subgroup slopes segregated only at the trend level for IL-6 over the 0-to-120 min period (Fig. 4c). Similar to RCA, heart rate and the HRV metrics showed limited significant slope differences between the high and low responder groups although it was apparent with: (1) TNF-α RMSSD and SAI (0-120- and 120-240 min interval respectively) and (2) IL-1β RMSSD and SAI (0-120- and 120-240 min interval respectively) (Fig. 5). LPS-elicited differences in clinical symptom score were evident between the high and low-responder subgroups for TNF-α and IL-1β. This significant difference between the two subgroups emerged at the 180-min time point. Based on subgroup differences in IL-8 levels, the difference in clinical symptoms was manifest at both the 180 min and 240-min time points (Supplementary Fig. 5). Comparisons between RNG and RCA sensor site (aggregate CAP firing frequency) in the High and Low responder subgroups based on IL-10 levels revealed that in the high responders: (1) the AUC tended to be distinct between 0-to-30-min and (2) there was a trend level difference in the slope of increase between RNG and RCA from 0-120 min (Supplementary Fig. 6).

### TNF-α temporal matching with ventral cervical magnetoneurography and cardiac autonomic measures

**Fig. 6:**
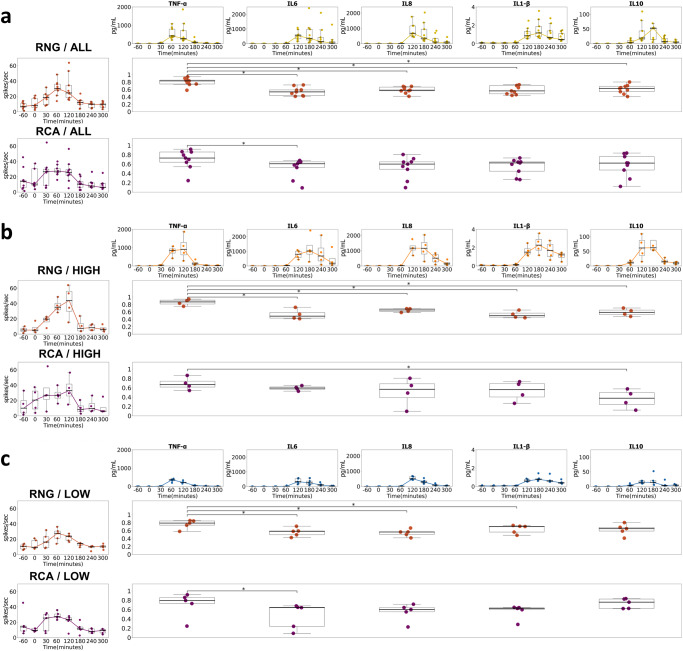
Temporal similarity between all cytokine concentrations and MNG neural activity for all subjects, high responder, and low responder subgroups. Normalized template matching was deployed to determine the temporal similarity between MNG derived (RNG and RCA) firing rate and each cytokine change over the 7-Hr protocol for all subjects (**a**), high responder subgroup (**b**), and low responder subgroup (**c**). The two-sided Wilcoxon Rank Sum test was employed to compare similarity scores between TNF-α and four other cytokines (IL-6, IL-8 and IL-1β, IL-10) (*=*p* < 0.05). Boxplot centerline: median; box limits: Q1 and Q3 quantile.

**Fig. 7:**
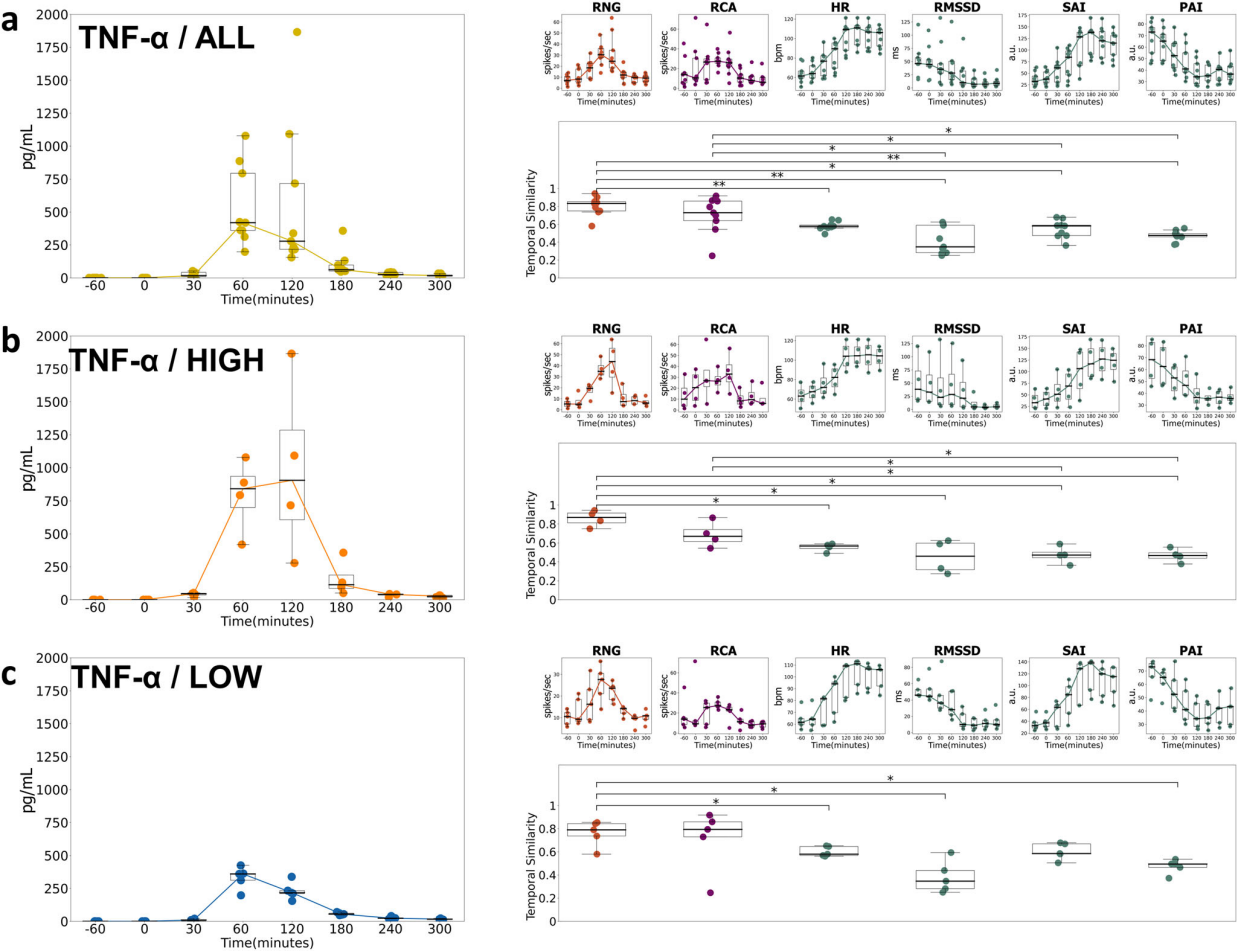
Temporal similarity between TNF-α and MNG and CAM. Normalized template matching was deployed to find the overall pattern of similarity between TNF-α changes and RNG, RCA, Heart rate, RMMSD, SAI, and PAI metric change for all subjects (**a**), TNF-α high responder subgroup (**b**), TNF-α low responder subgroup (**c**). The two-sided Wilcoxon Rank Sum test was employed to compare similarity scores between TNF-α (all, high and low) amongst RNG, RCA, and cardiac-related metrics (HR, RMSSD, SAI, PAI). (* *p* < 0.05, ** *p* < 0.01). Boxplot centerline: median; box limits: Q1 and Q3 quantile.

Estimates of temporal similarity between cytokine responses to both neural indices (vcMNG) and CAMs were analyzed with the normalized template matching method as described previously by Laboy-Juárez et. al. ^69^ (see Methods). In brief, normalized template matching compares the correlation of two vectors over time (i.e., the shape similarity), with values ranging between -1 (negative correlation) to +1 (positive correlation). Among the quantified cytokines, TNF-α had the highest pattern similarity with RNG and RCA firing rates (Fig. 6). When comparing all subjects and the high responders (Fig. 6a, b), we found that RNG activity was most similar to changes in TNFα levels when compared with all other cytokines (p < 0.05). The RNG sensor in the low responders also showed a higher concordance with TNF-α than with all other cytokines except for IL-10 (Fig. 6c**;** p < 0.05). The RCA also exhibited significantly greater normalized template-matched similarity with the TNF-α response when compared to IL-6 among both all subjects and low responders (Fig. 6a, c; p < 0.05), and as well as with IL-10 among the RCA high responders (Fig. 6b; p < 0.05). Based on this finding, TNF-α was selected as the exemplar cytokine for temporal similarity analyses of both neural indices and CAMs for all, high and low responders (Fig. 7). Across TNF-α all and high responders (Fig. 7a, b), RNG and RCA consistently demonstrated greater normalized template-matched similarity when compared to HR, RMSSD, SAI, and PAI (p < 0.05 – p < 0.01). In the TNF-α low responder subgroup (Fig. 7c), only the RNG normalized template matching remained significantly greater than CAMs, including HR, RMSSD, and PAI (p < 0.05). We observed that the RNG revealed greater temporal similarity with TNF-α compared to its association with HR, RMSSD, and SAI and PAI CAMs. The temporal similarity analyses for IL-6, IL-8, IL-1β, and IL-10 are presented in Supplementary Fig. 7. In aggregate, RNG CAP firing frequency provided an accurate proxy of the temporal trajectory of the TNF-α cytokine response, whereas CAMs temporally aligned more closely with the changes in IL-6, IL-8, IL-1β, and IL-10 levels (Supplementary Fig. 7).

## Discussion

The intravenous LPS challenge protocol provides a controlled model that recapitulates both the inflammatory and physiological responses observed in systemic inflammation and the acute endotoxemia that precedes clinical sepsis. Inflammatory reactions are multifactorial and include changes at the molecular, cellular, tissue, and organismal levels. The global response is regulated and moderated in large part by the neuroimmune axis^10,23,26,70–72^. To date, examination of the neuroimmune axis has relied largely on pre-clinical experiments, often requiring invasive procedures^19–21,48^. By employing non-invasive vcMNG (using wearable conformal OPMs) while evoking a large inflammatory response to LPS in healthy participants, we were able to detect ventral cervical neural firing that were correlated with peripheral blood cytokine changes pre-to-post LPS. These results serve as a proof-of-concept validation of recording technology and methodology, which are capable of non-invasive clinical monitoring of the neuroimmune axis in humans. Within our test cohort, we observed two distinct endotype subgroups who were low and high responders to LPS based on the peak titer of each cytokine. The work yielded four major findings. First, we observed significant early changes in the aggregate CAP firing frequency at the RNG site within 30 min of intravenous LPS injection. Second, the overall neurological measurements showed a strong correlation with the temporal changes in TNF-α levels, while CAMs were more correlated with the temporal trajectory of the changes in IL-6, IL-8, IL-10, and IL1-β. Third, the aggregate CAP firing frequency (AUC) at the RNG site segregated subgroups based on high and low IL-6 during the early 30-120 min interval. Fourth, RNG and RCA vcMNG neural firing evinced greater temporal similarity to the changes in TNF-α levels when compared to conventional CAMs, including SAI and PAI. In sum, the findings demonstrate the feasibility and potential clinical utility of vcMNG as a bioindicator of the human inflammatory response. Prospective assessment of the accuracy of the vcMNG-derived prediction would be of value when clinically monitoring an infected inpatient.

While the MEG-derived magnetic fields generated by brain neuronal ensembles are well established, this study is the first to refine a conformal sensor paradigm that captures changes in the magnetic field from the ventral cervical area using OPMs. Ganglia (i.e., nodose and jugular Ganglion and petrous ganglia) and sinus nerves (i.e., carotid sinus) at these sites are integral to transmitting and modulating afferent autonomic information^73,74^. The ability to capture functional changes in the vcMNG response to stimuli lays the foundation for inflammatory diagnostics and or development of closed-loop treatment modalities^75–77^. As the most widely utilized in vivo inflammatory challenge model, LPS induces cytokine synthesis and release at high systemic levels that cannot be readily recapitulated with cell cultures in vitro or even with ex vivo organ models^9,78^. When LPS binds to macrophage Toll-Like receptor 4 (TLR4), it activates the nuclear factor κB (NF-κB) inflammatory pathway, which leads to the synthesis of many different cytokines that can act synergistically or in the case of IL-4 and IL-10 moderate the actions of proinflammatory signaling proteins^79,80^. However, in some cases, the surging of IL-4 and IL-10 contributes to a deleterious anti-inflammatory innate immune phenotype known to result in immunoparalysis^81,82^. Inflammatory cytokines produced by immune cells during infection and injury activate sensory neurons (i.e., including C-fibers that are highly represented within the vagus nerve) in the form of CAPs^19,48,83–85^. In preclinical models, TLR4 has been detected in both the nodose ganglion^73^ and carotid body, a chemo-sensitive paraganglia^86^. Further RNA sequencing demonstrates capsaicin-responsive nodose and jugular ganglion neurons express interferon receptors that are responsive to IFN-γ, IFN-α, and CXCL8^87^. In line, sensory neural signals identified from the vagus nerve are cytokine-specific in murine models^19–21,48^. We noninvasively monitor vcMNG overlying autonomic ganglia including the nodose, jugular, the carotid sinus and distal vagus nerve at the carotid bulb in order to quantify aggregate neural firing frequency change indicative of afferent and or efferent autonomic neural signaling.

TNF-α, a key component of the inflammatory response, orchestrates the production of a cascade of other pro-inflammatory cytokines, making it an important initiator and regulator of cytokine responses^88,89^. TNF-α is released by many different immune cells, particularly macrophages and NK cells, in response to different stimuli, such as trauma, infection, or exposure to lipid A (or endotoxin), a non-repeating “core” oligosaccharide, and distal polysaccharide (or O-antigen) that in aggregate comprise LPS^90^. Its rapid release contributes to and shapes the early phases of inflammation^91^. Unique to our study, we observed changes in vcMNG activity post-LPS administration that were correlated and temporally concordant with both the rise and subsequent decline in TNF-α levels (Figs. 3, 6, 7). In the clinical setting, a care provider may need to track the efficacy of treatments that reduce peripheral inflammation. Future studies are now planned to determine if noninvasive vcMNG at the RNG site can be employed to detect the initial TNF-α rise and the subsequent decline and resolution in patients admitted with infectious disease. This information can be utilized to refine therapeutic interventions, i.e., either to continue or discontinue the treatment. Further, planned human infection challenge studies will dynamically track vcMNG and CAM features that have a high correlation with specific cytokines with the aim to enhance the clinician’s ability to distinguish and predict a pathogen specific inflammatory response including SARS-CoV-2^92,93^.

Exposure to acute stress and trauma in individuals with depression, anxiety, and posttraumatic stress disorder (PTSD) can amplify central fear appraisal systems that are known to increase cytokine release when exposed to stress^34–42^. These psychological states also reduce cholinergic and beta-adrenergic mediated inhibition of cytokine release^94,95^. Further, prior work has shown that anxiety disorders are associated with larger cytokine responses when challenged with LPS, both in vivo and in stimulated cell cultures^37,71,72,78,96^. In the current study of healthy participants, individual variation was examined by categorizing the participants into low and high responder subgroups based on peak pro-inflammatory cytokine concentrations (TNF-α, IL-6, IL-8, IL-1β) and the anti-inflammatory cytokine (IL-10). The slope of change in RNG was greater in high responders when delineated by TNF-α, IL-6, IL-8, and IL-1β at the early time point from 0-120 min. In contrast, when compared to the low responders, high responders based on TNF-α, IL-8, and IL-1β exhibited more severe sickness symptoms post-LPS at the later 180- and 240-min time points. It should also be highlighted that significant subgroup segregation via vcMNG preceded significant subgroup segregation via conventional sick symptom (i.e., at lower cytokine concentrations), indicating that vcMNG could be used as an early pre-symptomatic predictor of imminent inflammation^72^. Based on these findings, vcMNG recordings are planned in LPS-challenged general anxiety disorder and/or PTSD patients. Collectively, it may identify inflammation-mediated changes in vcMNG that can be used as early and sensitive bioindicators of neuroimmune axis dysregulation associated with mental health disorders.

The vagus nerve transmits sensory afferent information related to chemical, temperature, mechanical, noxious stimuli^97^ and cytokine concentration change^19,20,48^ while the nodose and jugular ganglion are known to express TLR4 receptors responsive to LPS^73^. Further, the nodose and jugular ganglion respond to incremental increases in vagus afferent CAP amplitude by directly increasing projection neuron firing frequency^74^. Put simply, as the afferent signal is amplified, vagus to brain stem signaling complexity increases^74^. In aggregate, the rostral nodose and jugular ganglion are sites of code multiplexing of peripheral afferent vagus signaling while also segmentally responsive to LPS^73–75,97,98^. When comparing rostral RNG to caudal RCA aggregate neural firing of the low and high subgroups, not all cytokines revealed site specificity (Supplementary Fig. 6). However, the rostral RNG sensor site showed neural responsivity that aligned to cytokine concentration change; it was more aligned to TNF-α when compared to the caudally positioned RCA sensor site. Further, the RNG sensor site (aggregate CAP firing frequency) subgroups significantly segregated IL-6 as early as the 30-60 min interval and showed trend differences for the remaining cytokines except for IL-10. Similarly, the RNG sensor site slope (aggregate CAP firing frequency over early and late protocol periods i.e., 0–120 and 120–240 min) significantly segregated IL-6 and segregated all other cytokines at the trend level except for IL-10. In contrast to the RNG site, the RCA sensor site aggregate firing rate significantly segregated groups for IL-10 and IL-6 at the 0–30- and 180–240-min intervals, respectively, while no significant slope subgroup differences were detected (Fig. 4). Collectively, these data indicate an overall higher sensitivity of both RNG and RCA to IL-6 and an earlier sensitivity of the RNG sensor site to inflammatory cytokines when compared to the RCA sensor site.

Clinical sepsis related immunoparalysis is known to present with an anti-inflammatory innate immune phenotype that is hallmarked by increases in IL-10 concentrations^81,82^. At the RCA sensor site, early IL-10 sensitivity is suggestive of a potential site-specific bioindicator of IL-10 dysregulation that may be leveraged in future studies providing a novel surrogate marker for immunoparalysis in the clinical setting. Sympathetically mediated peripheral noradrenergic drive acutely attenuates pro-inflammatory cytokine release^99^ but is also known to enhance levels of IL-10^10,99^. Identification of site-specific RNG vs RCA firing frequency change may provide a proxy of intrinsic noradrenergic drive during an LPS challenge, and more broadly may generalize to identification of autonomic endotypes in future clinical studies. In aggregate, the RNG sensor site tended to exhibit higher sensitivity to the inflammatory cytokine TNF-α than the RCA site, while the RCA sensor site may exhibit a higher sensitivity to the anti-inflammatory cytokine IL-10. Future studies will parse out site-specific LPS induced cytokine to neural encoding with planned invasive probe (ultrasound-guided microneurography) techniques at each site^100^; it may further disentangle site-specific cytokine signaling.

The primary strength of this study was the implementation of a standardized and reproducible experimental model of the human endotoxin challenge (i.e., intravenous LPS injection) which permitted continuous neuroimmune axis monitoring in a controlled setting. Nevertheless, several limitations should be acknowledged. First, this study is limited by the relatively small number of subjects that were then divided into high and low subgroups. Although the sample size is small, within-subject sampling times were quite dense (ms recordings), which substantially increases the meaningfulness and reliability of the identified groups of the subjects. Larger studies are planned in which we will better segregate high and low subgroup endotypes. Second, the participants were all male and healthy. It is known that there are significant differences in the cytokine responses to LPS between men and women^101^ while CAM is also modulated by the female estrous cycle^102^. Genetic, psychological, sleep duration and other physical factors impel the cytokine response to LPS injection^103^, and the impact of those factors could not be considered in our analyses. Future LPS challenge studies are planned to recruit both female and male participants as well as patients with PTSD, major depression disorder and clinical insomnia. Third, due to feasibility and ethical considerations, we collected peripheral blood at 0.5-to-1 h intervals. For a more fine-grained quantification of temporal changes in cytokines, MNG and autonomic measures, wearable patch sensors may be employed in the future enabling more continuous assessment. To achieve this goal, flexible microneedle electronics to amperometrically calculate cytokine on-demand concentrations are planned^104^. Fourth, although more invasive methods could allow us to distinguish and source localize nodose and jugular ganglion versus caudal vagal nerve and carotid sinus activity, this first feasibility study was designed primarily to employ signal acquisition methods that could be implemented in a clinical setting. With further advances in noninvasive methods for acquiring neurological signals^105^, an opportunity to validate human nerve B-field signals in a relatively safe and reversible manner, is now attainable. Future work will distribute OPM arrays bilaterally over the ventral cervical area with the aim to distinguish laterality and source localize the current dipole. Further these comparisons would allow noninvasive nerve measurements to detect body site-specific (i.e., arrays placed over sympathetic chain, or peripheral organ targets such as splenic nerve) MNG change more systematically throughout the peripheral nervous system. Convex optimization methods to quantify heartbeat dynamics^106^, Empirical Mode Decomposition, Independent Component Analysis, and Spectral Interpolation will be deployed in future studies to refine and improve the signal-to-noise ratio.

In summary, this work identified a new and highly sensitive bioindicator of the neuroimmune axis: ventral cervical neural MNG. The vcMNG response acquired at 30 min after LPS administration temporally aligned with the changes in TNF-α levels, both the rise and the subsequent decline, more specifically than available CAMs. Post-hoc analyses of the cytokine responses of different individuals identified endotype subgroups that segregated RCA and RNG CAP frequency at the early 0-30- and 30-120-min intervals for IL-10 and IL-6, respectively. Therefore, the tools for acquiring vcMNG may be deployed to anticipate and categorize inflammatory profiles in patients admitted with infection or injury.

## Methods

### Participants and experimental setup

The Institutional Review Board at the University of California, San Diego Health Systems reviewed and approved the experimental protocol (UCSD IRB #191463). Twelve physically healthy adult males, without signs or symptoms of infection gave their written consent prior to study procedures. All ethical regulations relevant to human research participants were followed. Within 12 h prior to their visit, all subjects were kept fasted and told to refrain from showering or bathing. Subjects were asked to refrain from ingesting any caffeinated products, tobacco, or alcohol for 24 h prior to the protocol. The study was carried out within the UCSD Radiology Imaging Laboratory’s six-layer magnetically shielded room (MSR) (IMEDCO, Switzerland) to minimize the effects of powerlines and Earth’s magnetic field on sensor signals. The MSR has a shielding factor of 65–160 dB for the 0.01 Hz–10 Hz frequency range. Each participant was asked to remove all electronic equipment and metal accessories and to wear a hospital gown prior to entering the MSR to avoid magnetic noise and sensor saturation. The study started at 07:30 AM, and subjects were kept (nothing per oral) NPO during the test session. All subjects received 1 L of 0.9% sodium chloride intravenously 1 h prior to LPS injection (Fig. 1). Participants were seated in an adjustable plastic chair (Elekta-Neuromag, Sweden) centered within the MSR. Subjects were allowed to select and watch a feature movie from any genre except horror during the study.

### LPS intravenous injection

Participants received the 3 ng/kg LPS intravenous injection followed by a 10 ml normal saline flush. LPS (US Standard Reference Endotoxin derived from Escherichia coli O:113) obtained from List Biologics (Lot #94332B4) was supplied as a lyophilized powder. It was reconstituted in 2 mL sterile 0.9% saline solution for injection after which it was vortexed for 15 min. A professional infusion-center (UCSD Moore’s Cancer Center) oncologic nurse inserted two large-bore (14 gauge) catheters into the median cubital vein localized in the antecubital fossa 30 min before the start of the trial. Catheters were used for blood draws and continuous infusion of 0.9% NaCl normal saline. 0.9% NaCl normal saline was administered intravenously by an Alaris IV infusion Pump (BD Technologies and Innovations, NJ, USA). Post LPS injection, 0.9% NaCl normal saline was administered at a rate of 450 mL/h for 1 hr and then continued at 100 mL/h for the remainder of the test protocol^107^.

### Blood draw, symptom, and blood pressure collection

Peripheral blood draws were performed via intravenous catheter at one Hr prior to injection (–60 min), immediately before LPS injection (0 min), as well as 30-, 60-, 120-, 180-, 240-, and 300-min post-LPS injection (Fig. 1). A strict sterile technique was employed for all blood draws. Blood was centrifuged, plasma collected in 0.5 ml aliquots, and immediately stored (within 15 min) at –80 °C. At the time of the blood draw, subject symptom severity was measured using self-reported symptoms as per the standard Likert scale (range; 0–5, none to extreme discomfort) for headache, nausea, rigor, and myalgia^24^.

### Cytokine analysis

Plasma aliquots were analyzed with a multi-cytokine array using an electrochemiluminescence platform and concentrations were quantified on a multiplexing MESO QuickPlex SQ120 for analyte detection (Meso Scale Discovery, Gaithersburg, MD). The panel included interferon-gamma (IFN-γ), interleukin-2 (IL-2), interleukin-12p70 (IL-12), interleukin-13 (IL-13), interleukin-4 (IL-4), interleukin-1beta (IL-1ß), interleukin-6 (IL-6), interleukin-8 (IL-8), tumor necrosis factor-alpha (TNF-α), and interleukin-10 (IL-10). All samples from each participant were run in duplicate determinations on the same assay plate to ensure identical quantification of the 8 serial specimens. The same 7 calibrators with cytokine standards of known concentration were included on the 5 plates used to assay all specimens in order to generate consistent reference curves calculated from the 7 standards. The lower limit of quantification for maximal accuracy (LLOQ) and lower limit of detection (LLOD) for the MSD platform for the 5 cytokines included in this report were: IL-1β: 0.14, 0.03; IL-6: 0.22, 0.11, IL-8: 1.13, 0.16; IL-10: 0.08, 0.02; TNFα: 0.31,0.10 pg/mL. The MSD platform offers a wide dynamic range allowing cytokines to be quantified at very low picogram level as well as high picogram level. Notably, at the high picogram level the linear portion of the reference curve was observed when reaching several hundred picograms during peak response to the endotoxin challenge, between 1 and 4 h. All serial samples for each participant were processed in the same assay. Further, the serial responses after the LPS injection were compared to baseline levels, employing a within-subject analysis to strengthen the reliability of detecting sustained increments or decrements in cytokine levels. Only IFN-γ, IL-12, IL-13, IL-4, IL-1ß, IL-6, IL-8, TNF-α, and IL-10 were reported as the IL-2 assay demonstrated a repeated aberrant outlier measure.

### Ventral Cervical OPM

OPMs are an emerging class of quantum magnetic sensors with a demonstrated sensitivity at 1 ft/√Hz; they are capable of detecting changes in cortical and peripheral neuronal currents as previously described by our group^43^. Each OPM sensor contains a glass vapor cell with enclosed rubidium atoms. The glass vapor cell receives circularly polarized laser light directed by a prism towards a photodetector to monitor the light intensity transmitted through the vapor cell^108^. When the background magnetic field is equal to zero, the circularly polarized laser light spin polarizes rubidium atoms in the direction of the light beam, making the rubidium atom transparent to the incoming light^109^. A magnetic field in the direction perpendicular to the light path causes the rubidium atoms to absorb light. The photodetector identifies the change in transparency and, as such, measures voltage as a function of the external magnetic field, for review see^110,111^. Commercially available QuSpin Gen-2 OPM (QuSpin Inc., CO, United States) sensors are conformal with a 6.5 mm sensor stand-off, while our group recently modified the OPM to extend the bandwidth up to 500 Hz at a sensitivity of 20 ft/√Hz^43^. OPMs were adhesively attached to both ventral cervical at the RNG and RCA sites. Adhesion was accomplished by applying the loop side of the adhesive Velcro strap to the skin surface above the Right Nodose Ganglion (RNG) and Right Carotid Artery (RCA), while the hoop side was attached on the OPM x-axis surface. The x-direction of the OPM was adjusted to be normal to the skin surface in the longitudinal direction along the vagus nerve and carotid sinus. To improve sensor to target distance each OPM was further secured with medical adhesive tape (3 M Microfoam Surgical Tape) applied to both sensor and skin.

Prior work from our group demonstrated OPM sensory nerve action potential (SNAP) measurement from the human median nerve at the forearm and upper axilla^43^. The OPM acts as a single sensor with a fixed effective distance of 6 cm (i.e., circumferential diameter/sensor field at which the median nerve propagating action current is detected). The OPM has a 6.5 mm sensor stand-off, while the depth of target structures (vagus nerve and carotid sinus at the cervical #4 level) ranged from 10 to 15 mm as measured with a portable ultrasound transducer (Butterfly IQ, Palo Alto, United States). At the angle of the mandible, the depth of the distal nodose ganglion (in close approximation to the internal jugular vein and internal carotid artery at the cervical #1-2 level) was identified at 10–13 mm with portable ultrasound imaging. Taken together, the vagus nerve, carotid sinus nerve, and nodose ganglion are well positioned within the OPM measurement radius (effective distance prior demonstrated).

The Sarvas formula was employed to estimate magnetic field measured at the ventral cervical sites. The Sarvas formula, an analytical Biot-Savart law solution is deployed to evaluate magnetic field at the sensor site by introducing the concept of current dipole and assuming a spherically symmetric conductor^112^. Though the cervical structure is cylindrical rather than spherical (utilized for brain MEG), the Sarvas formula is effectively calculated as the cervical source and OPM sensor lie on a concentric circle. The anatomical distance between the circle center (center of axial cervical segment) was confirmed with cervical MRI and transverse ultrasound imaging; the source (vagus nerve/carotid sinus) was ~40 mm from the circle center, while the distance between the circle center to sensor was 52 mm while we added the QuSpin 6.5 mm sensor standoff to reach 58.5 mm. We deployed the current dipole moment formula introduced by Hämäläinen et al. ^113^, Eq. (1).

1 $${{{{\rm{Q}}}}}={{{{\rm{\pi }}}}}{{{{{\rm{d}}}}}}^{2}{{{{{\rm{\sigma }}}}}}_{{{{{\rm{in}}}}}}\Delta {{{{\rm{V}}}}}/4$$

where d is the diameter of the axon, *σ*_*in*_ is the intracellular conductivity, and ΔV is the action potential amplitude. 1 Ω^-1^m^-1^ was chosen as σ_in_ from Hämäläinen et al. ^113^ Vagus fiber diameter and number of fibers were extrapolated from prior human dissection Hoffman et al. ^58^ of the right-sided vagus nerve, and swine vagus nerve macroscopic fascicular structure, effective nerve diameter, and fiber type decomposition^114–116^. Prior work demonstrate consistent action potential amplitude for A and C fibers at 70 mV and 80 mV respectively^74^. Current dipole moments *from both* myelinated and unmyelinated fibers were summated to calculate a combined current dipole moment of 70 nAm. In aggregate, the Sarvas formula resulted in a maximum measure of ~9 pT (if all axons activated). The magnetic field measured in all subjects (within the Sarvas formula calculation) consistently ranged from 1-4 pT (Supplementary Figs. 2, 3).

In aggregate, there are innumerable neural structures that contribute to the measured magnetic field at the ventral cervical surface. Unmyelinated C-sympathetic efferent fibers with a diameter ranging from 0.2 to 1.4 μm are known to activate with autonomic challenge (including cold pressor test, or pathogen challenge or infectious status)^117,118^. Minimal effects on measurable magnetic field are expected see equation from Hämäläinen et al. ^113^ due to the small diameter of cutaneous C-fibers^119^.

### Data recording and preprocessing

Each site (RNG and RCA) OPM were operated in single y-axis mode per pre-set configuration with the gain set to 0.9 V/nT. All OPM sensor heater frequencies were synced to 400 kHz to avoid beat-note frequencies between sensors. The physiological analog signal from the OPM electronic module was sampled by a CED Micro1401 device at 10 kHz and recorded by Signal 8.19a software (Cambridge Electronic Design, Cambridge, UK). OPM MNG recording sessions were paused and restarted between each blood draw to prevent OPM saturation. Prior to the initiation of OPM MNG recording, each OPM was tuned and calibrated in an environment within the sealed MSR. The power-spectrum density of the OPM noise level was ensured to be consistent under 500 Hz across all subject recording sessions. OPM measurement (B-field) reliably detects the QRS complex of heartbeats (8-50 Hz)^120^, respiration ( < 0.5 Hz)^121^, and electromyographic activity with muscle contraction (8-10 Hz)^122^. To minimize extraneous CAM, respiratory, and myographic physiological artifacts, a 50-500 Hz zero-phase bandpass filter was applied. Notch filters applied at multiples of 60 Hz removed the power line noise. MNG recording for each session was then concatenated for running the spike sorting algorithm to differentiate detected spikes into different clusters.

### Spike detection

Spike detection was performed by amplitude thresholding after filtered data adapted from works by Quiroga et al., and defined by Eq. (2) and Eq. (3) :

2 $${{{{\rm{Threshold}}}}}=3{{{{\rm{\sigma }}}}}$$

3 $${{{{\rm{\sigma }}}}}={{{{\rm{median}}}}}(\frac{|{{{{\rm{x}}}}}|}{0.6745})$$

*x* is the 50-500 Hz bandpass filtered data, and using a non-causal filter to filter data is critical because causal filters not only distort the spike shapes but can also change the appearance of artifacts and make them look similar to real neural data^123,124^. *σ* is an estimate of the standard deviation of the background noise as prior described by Quiroga et al. ^124^.

### Heart rate variability

RR intervals are canonically calculated as time measured in ms between each R peak of the QRS complex. QRS peaks were directly extracted using the Matlab R-DECO package from the entire 7 h session as previously described^125^. RR intervals were employed to calculate root mean square of successive RR intervals (RMSSD), sympathetic activity index (SAI), and parasympathetic activity index (PAI). SAI and PAI were estimated through Laguerre expansion of RR interval series, as described in^66^. Let *RR*_*k*_ denote the *k*^*th*^ RR interval, then the *j*^*th*^*-*order discrete-time orthonormal Laguerre function is defined as Eq. (4):

4 $${{{{{\rm{\phi }}}}}}_{{{{{\rm{j}}}}}}\left({{{{\rm{n}}}}}\right)={{{{{\rm{\alpha }}}}}}^{\frac{{{{{\rm{n}}}}}-{{{{\rm{j}}}}}}{2}}{\left(1-{{{{\rm{\alpha }}}}}\right)}^{\frac{1}{2}}\,{\sum }_{{{{{\rm{i}}}}}=0}^{{{{{\rm{j}}}}}}{\left(-1\right)}^{{{{{\rm{i}}}}}}\left(\begin{array}{c}{{{{\rm{n}}}}}\\ {{{{\rm{i}}}}}\end{array}\right)\left(\begin{array}{c}{{{{\rm{j}}}}}\\ {{{{\rm{i}}}}}\end{array}\right)\,{{{{{\rm{\alpha }}}}}}^{{{{{\rm{j}}}}}-{{{{\rm{i}}}}}}{\left(1-{{{{\rm{\alpha }}}}}\right)}^{{{{{\rm{i}}}}}}$$

with n ≥ 0 and *α* (the constant of decay) set to 0.2. First, the RR interval series is convolved with the below functions, as shown in Eq. (5):

5 $${l}_{j}\left(k\right)={\sum }_{n=0}^{k-1}\,{{{{{\rm{\phi }}}}}}_{j}\left(n,{{{{\rm{\alpha }}}}}\right){RR}\left(k-n-1\right)$$

Then, a theoretical autoregression model that can be used to separate sympathetic and parasympathetic dynamics, as Eq. (6):

6 $${{{{{\rm{\mu }}}}}}_{{{{{\rm{RR}}}}}}\left({{{{\rm{k}}}}},{{{{{\rm{H}}}}}}_{{{{{\rm{k}}}}}},{{{{\rm{\xi }}}}}\left({{{{\rm{k}}}}}\right)\right)={{{{{\rm{g}}}}}}_{0}\left({{{{\rm{k}}}}}\right)+\underbrace{{\sum }_{{{{{\rm{j}}}}}=0}^{{{{{{\rm{P}}}}}}_{{{\mbox{Symp}}}}}{{{{{\rm{g}}}}}}_{1,{{{{\rm{j}}}}}}\left({{{{\rm{k}}}}}\right){{{{{\rm{l}}}}}}_{{{{{\rm{j}}}}}}\left({{{{\rm{k}}}}}\right)}_{{{\mbox{Sympathetic}}}}+\underbrace{{\sum }_{{{{{\rm{j}}}}}={{{{{\rm{P}}}}}}_{{{\mbox{Symp}}}}+1}^{{{{{{\rm{P}}}}}}_{{{\mbox{ParSymp}}}}}{{{{{\rm{g}}}}}}_{1,{{{{\rm{j}}}}}}\left({{{{\rm{k}}}}}\right){{{{{\rm{l}}}}}}_{{{{{\rm{j}}}}}}\left({{{{\rm{k}}}}}\right)}_{{{\mbox{Parasympathetic}}}}$$

Where ${{{{\rm{\xi }}}}}\left(k\right)={\left[{g}_{0}\left(k\right),{g}_{{{{\mathrm{1,0}}}}}\left(k\right),\ldots ,{g}_{1,j}\left(k\right)\right]}^{{{\top }}}$ are unknown time-varying Laguerre coefficients and are modeled according to dynamic systems that fulfill Eq. (7) and Eq. (8):

7 $${{{{\rm{\xi }}}}}\left(k\right)={{{{\rm{\xi }}}}}\left(k-1\right)+{{{{{\rm{\varepsilon }}}}}}_{{{{{\rm{\xi }}}}}}\left(k\right)$$

8 $${RR}\left(k\right)={l\left(k\right)}^{{{\top }}}{{{{\rm{\xi }}}}}\left(k\right)+{{{{{\rm{\varepsilon }}}}}}_{{RR}}\left(k\right)$$

${\varepsilon }_{\xi }\left(k\right)$ is the state noise with Covariance matrix *S*_*ξ*_ and ε_*RR*_(*k*) is the observation noise with variance *S*_*RR*_. The Laguerre coefficient *ξ*(*k*) can be readily estimated using Kalman filter with the time-varying observation matrix.

Finally, the definition of the SAI and PAI as a combination of disentangling Laguerre coefficients Ψ_*S*_ and Ψ_*P*_ is shown as Eq. (9) and Eq. (10):

9 $${SAI}\left(k,{{{{\rm{\xi }}}}}\left(k\right)\right)=\left[{\Psi }_{{S}_{0}}+{\sum }_{j=1}^{2}{\Psi }_{{S}_{j}}{g}_{1,j-1}\left(k\right)\right]/{RR}{\left(k\right)}^{2}$$

10 $${PAI}\left(k,{{{{\rm{\xi }}}}}\left(k\right)\right)=\left[{\Psi }_{{P}_{0}}+{\sum }_{j=1}^{7}{\Psi }_{{P}_{j}}{g}_{1,j+1}\left(k\right)\right]2{RR}\left(k\right)$$

*Ψ*_*S*_ and *Ψ*_*P*_ are the generalized values for the sympathetic and parasympathetic kernels, which were previously derived through a multiple regression analysis on data involving selective autonomic blockade during postural changes^66,126^.

$$\begin{array}{c}{\Psi }_{S}=\{39.2343,10.1963,-5.9242\}\\ {\Psi }_{p}=\{28.4875,-17.3627,5.8798,12.0628,5.6408,-7.0664, -5.6779,-3.9474\}\end{array}$$

### Repeated measure correlation

To compare pair-wise correlations among neurological (i.e., RNG, and RCA neural firing), physiological (i.e., CAMs), and biological (i.e., cytokines) features, we first converted continuous measures (i.e., neurological and physiological) to discrete representative values at each blood draw time point to match cytokine measures. The average measurement for 5 minutes before and 5 minutes after the blood draw was selected as the representative value at that time point for each metric. The baseline (-60 min) measurement was calculated using an average of 10 minutes of activity post-baseline blood collection because the data acquisition had not started before the first blood draw. Then, repeated measure correlation (rmcorr) was computed between each pair of features with each individual as repeated measures. Unlike standard correlation/regression techniques, rmcorr can handle repeated measures data without violating independence assumptions or requiring first average the data. Therefore, rmcorr is ideal for accessing a common association across individuals, specifically a homogenous intra-individual linear association relationship between two paired measures^68^. All p-values have been corrected for multiple comparisons using the Benjamini-Hochberg method.

### Normalized template matching

We utilized normalized template matching described by Laboy-Juárez et. al to find pattern similarities between cytokine concentration change and physiological metrics^69^ for each subject. The method was proposed to find correlations of two vectors in time (i.e., shape similarities) ranges between -1 (negative correlation) to 1 (positive correlation). Here, we define the cytokine concentration change as a time-dependent row vector U(t) = [u(t)_-60_, u(t)_0_, u(t)_30_, …, u(t)_300_], where u(t)_T_ is the cytokine level from the blood draw at time T. Similarly, the time-dependent neural or physiological change vector is defined as V(t) = [v(t)_-60_, v(t)_0_, v(t)_30_, …, v(t)_300_], where v(t)_T_ is the average of 10 mins data after each blood draw at time T. The normalized template matching similarities is defined as Eq. (11)

11 $$S=\frac{U(t)\cdot {V(t)}^{T}}{{{{{\rm{||}}}}}U(t){{{{\rm{||\; ||}}}}}{V(t)}^{T}{{{{\rm{||}}}}}}$$

Where ${(\cdot )}^{T}$ is the vector transpose and ${||}\cdot {||}$ is the vector amplitude. The dot product between *U*(*t*) and *V*(*t*) can be rewritten as Eq. (12)

12 $$U(t)\cdot {V(t)}^{T}={{{{\rm{||}}}}}U(t){{{{\rm{||\; ||}}}}}{V(t)}^{T}{{{{\rm{||}}}}}\cos (\theta )$$

Where *θ* is the angle between vectors *U*(*t*) and *V*(*t*). Therefore, the normalized template matching is equivalent to cosine similarity between two vectors.

### Statistical analyses

The statistical comparisons of each metric post LPS to baseline levels were separated for discrete measures (i.e., cytokine concentration levels) and continuous measurements (RNG, RCA neural firing, and CAMs) (Fig. 2). Cytokine levels, which were sampled at each blood draw, were first evaluated to address multiple comparisons with an omnibus test to confirm overall main effect of time regardless of whether the homoscedasticity holds true and then compared by the two-sided paired t tests between cytokine levels at each time point with respect to baseline (Fig. 2d). To compare the mean level of continuous measurements (RNG, RCA, heart rate, RMSSD, SAI and PAI) between the baseline session (from –60 to 0 time point; at which LPS is injected) and post-LPS injection (between each blood draw 0–30 min, 30–60 min, 60–120 min, 120–180 min,180–240 min, and 240–300 min), we employed generalized estimating equations (GEE), where: (1) time (continuous), (2) session (multiple binary indicators with baseline session as the referent) and (3) their interactions were the predictors of each continuous variable. The semi-parametric GEE posits no mathematical model on data distribution such as normality and thereby provides valid inference for a broad class of data distributions^127^. Statistical inference was based on the score-like test (p-value) of GEE to improve inference validity (Fig. 2a–c).

MNG (RNG, RCA) and CAM recording time for each full session varied due to requisite restroom requests from each subject and or time required to obtain viable peripheral blood sample. To minimize variability of MNG and CAM recordings between subjects, we compared the average 10-min after the beginning of each session as the representative value for each time point (Fig. 2a, b, Fig. 4b, c, Figs. 5–7, Supplementary Figs. 6, 7).

To compare the differences between low and high responders, the 7-Hr session was divided into 7 sessions defined as the recording period between each blood draw. Two statistical approaches were performed. First, GEE was used to compare the cumulative change as evaluated by the Area Under the Curve (AUC) between the two subgroups during each session (1-7), where subgroup (a binary indicator), phase (multiple indicators with phase -60 to 0 as the referent) and their interactions as the predictors. Second, 0-120-min and 120-240-min time intervals were designated as increasing and decreasing MNG and CAM change periods. Differences in slope at across phase between subgroups were tested by score-like test of GEE on the interaction between time (continuous) and subgroup (a binary indicator) (Fig. 4b, c, and Fig. 5). AUC difference and the slope difference between RNG and RCA were tested by the same approaches (Supplementary Fig. 6).

### Reporting summary

Further information on research design is available in the Nature Portfolio Reporting Summary linked to this article.

## Data availablity

The source data used to generate graphs in the paper can be found in the Supplementary Data File.

### Supplementary information

Supplementary Information

Description of Additional Supplementary Materials

Supplementary Data

Reporting summary

## Data Availability

No custom code was used to generate or process the data described in the manuscript.
