# Supplementary material for: Non-invasive ventral cervical magnetoneurography as a proxy of in vivo lipopolysaccharide-induced inflammation
Source: Commun Biol. 2024 Jul 29;7:893. doi: 10.1038/s42003-024-06435-8 (PMC11286963; doi:10.1038/s42003-024-06435-8)
Supplement: Supplementary file 1 — Supplementary Information [file 42003_2024_6435_MOESM1_ESM.pdf]

Demographic	
Age	21.8 ± 4.1
BMI	22.86 ± 4.95
Race or ethnic group - no.	
Caucasian	6
Asian	3
Hispanic	2
SF-MPQ-2	5.1 ± 6.2

Supplementary Table 1: Demographic information for all subjects*. All continuous variables are formatted as Mean ±Standard Deviation. SF-MPQ-2 - Short-Form McGill Pain Questionnaire 2: This 22-question questionnaire is used to measure the quality as well as the intensity of pain on a scale of 0 (no pain) to 10 (extreme pain) for each descriptor. The sum of all descriptors is taken as the total pain inventory (maximum value: 22); lower values indicate less pain. All subjects' ratings were within the range of a healthy, reasonable pain scores and were thus included in the analyses.*

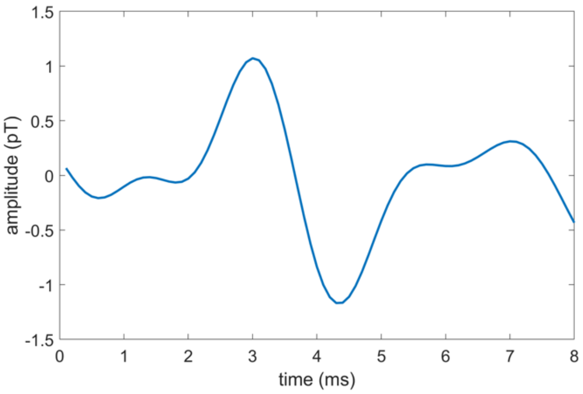

***Supplementary Figure 1**: An exemplary spike waveform captured by an OPM and classified using the spike detection algorithm*. *Our sampling rate of 10 kHz allowed us to sample 10 times per millisecond (ms), generating an 80-sample array for each 8-ms waveform. All waveforms are aligned by setting their maximum value (peak) to be located at the 3 ms location.*

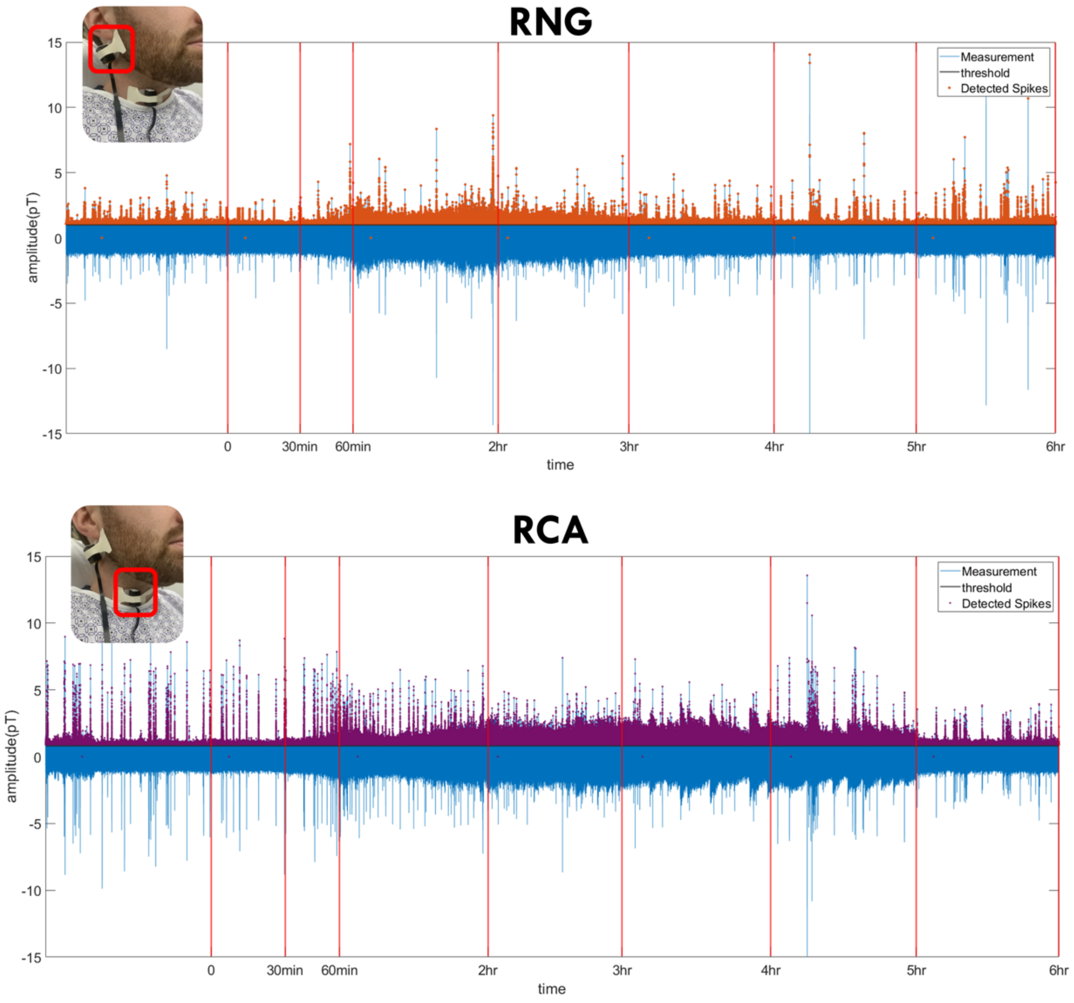

Supplementary Figure 2: Exemplary RNG and RCA MNG activity over the course of recording (-60 minutes through 6 hours) from an exemplary LPS subject. *Images of a subject in the top left corner of each sub-figure showed the location on the body where the RNG and RCA sensors were placed. The peak of each detected spike that exceeded the constant threshold (See Methods for details) was identified by different colors depending on the cluster with which it was classified. Vertical lines indicate the times of the serial blood sample collection. The amplitude of the MNG and the rate of occurrence of different clusters of spikes vary over the course of the trial. Vertical lines indicate the times of the serial blood sample collection.*

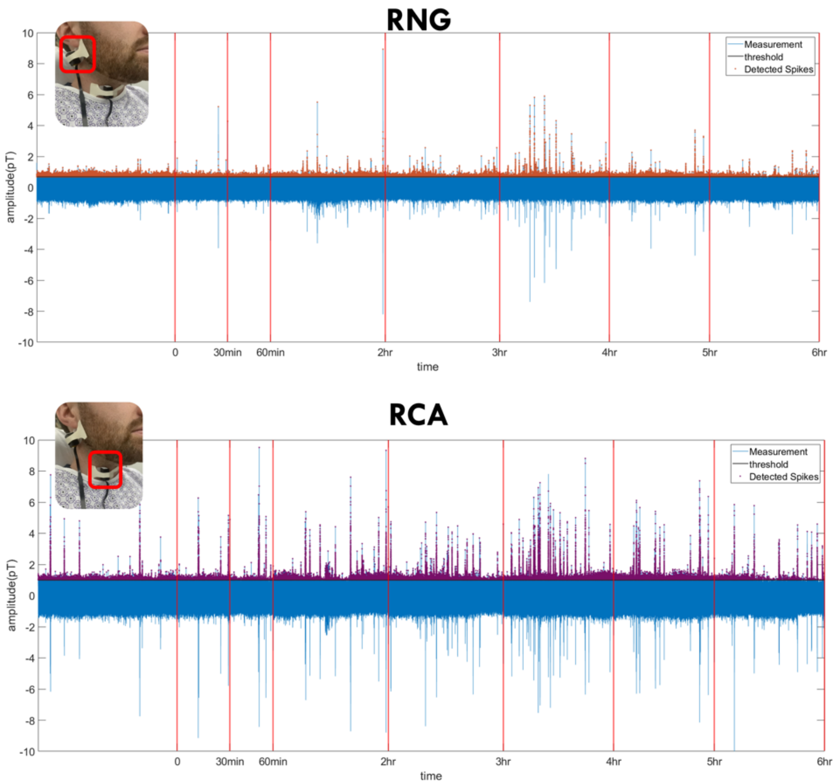

Supplementary Figure 3: Exemplary RNG and RCA MNG activity over the course of recording (-60 minutes through 6 hours) from a healthy control*. The healthy control follows the same protocol except without LPS injection. Images of a subject in the top left corner of each sub-figure showed the location on the body where the RNG and RCA sensors were placed. The peak of each detected spike that exceeded the constant threshold (See Methods for details) was identified by different colors depending on the cluster with which it was classified.*

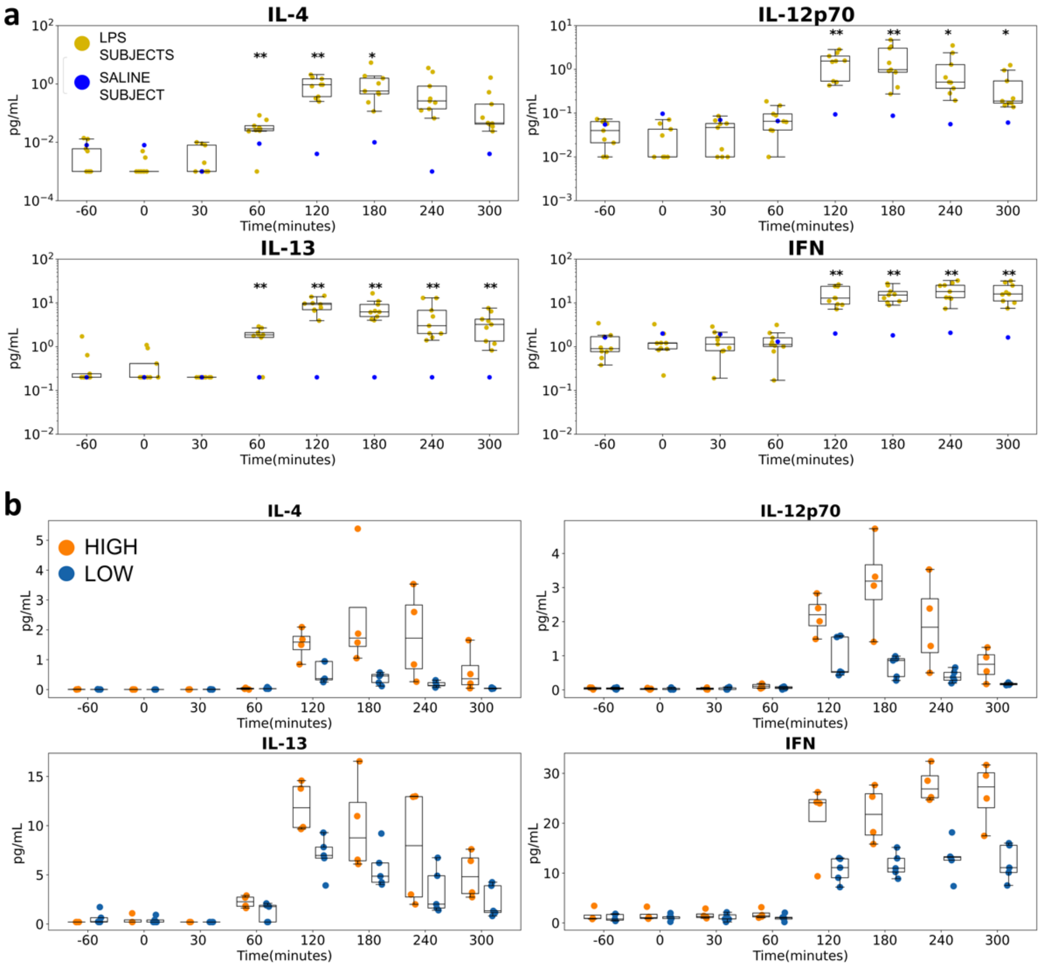

***Supplementary Figure 4: Concentration change in IL4, IL-12p70, IL-13, and IFN-ϒ pre-to-post LPS injection and high and low subgroups segregation. Panel A**: Changes in IL4, IL-12p70, IL-13, and IFN-ϒ concentration levels from pre-to-post LPS injection. Cytokine responses were first evaluated with an omnibus test to confirm the overall main effect of time and then paired t-tests were performed between each post-LPS time point and baseline. Significance was labeled with asterisk symbols above each bar (* $p<0.05$, ** $p<0.01$). The levels of IL-4, IL-12p70, and IL-13 were significantly increased at the 60-minute time point. Both IL-12p70 and IL-13 remained elevated until the 300-minute time point, while IL-4 returned to baseline levels at the 240-minute time point. IFN-ϒ was significantly increased at the 120-minute time point and a decrement was not evident until final 300-minute time point. **Panel B**: High and Low groups were shown with orange and blue bars, respectively for IL-4, IL-12p70, IL-13, and IFN-ϒ. Boxplot centerline: median; box limits: Q1 and Q3 quantile.*

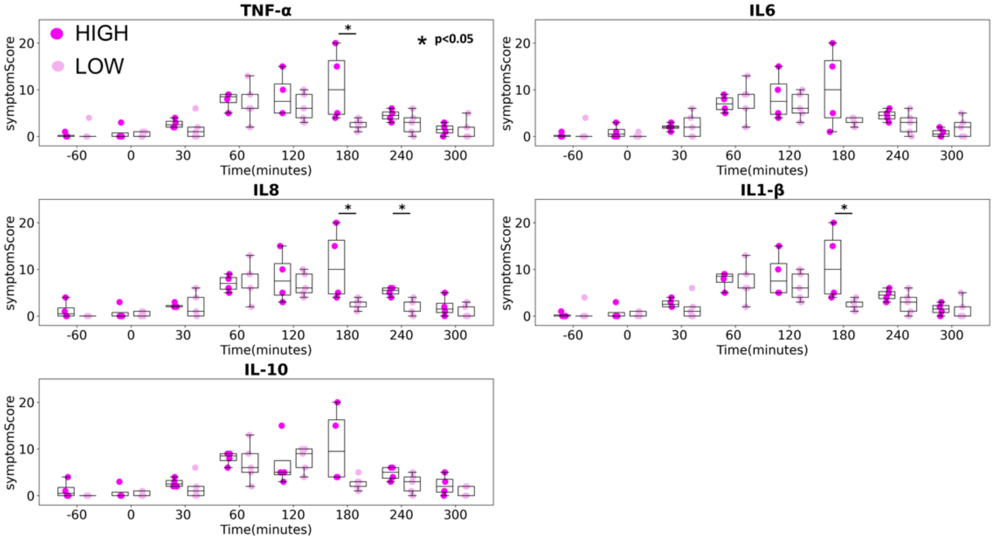

Supplementary Figure 5: Self-reported symptom scores between high and low responder subgroups for each cytokine*. The symptom questionnaire tracks headache, nausea, chills, and back pain severity using a self-reported system ranging from 0 (no feeling) to 5 (extremely discomforting). Statistical differences were assessed with the Wilcoxon Rank Sum tests. Significant differences in the association of symptoms with cytokines for the two groups were evident at the 180-minute time point for TNF-α and IL-1β. The two subgroups also evinced significantly different associations between symptoms and IL-8 at the 180- and 240-minute time points. No symptom differences were linked to IL-10 and IL-6. Boxplot centerline: median; box limits: Q1 and Q3 quantile.*

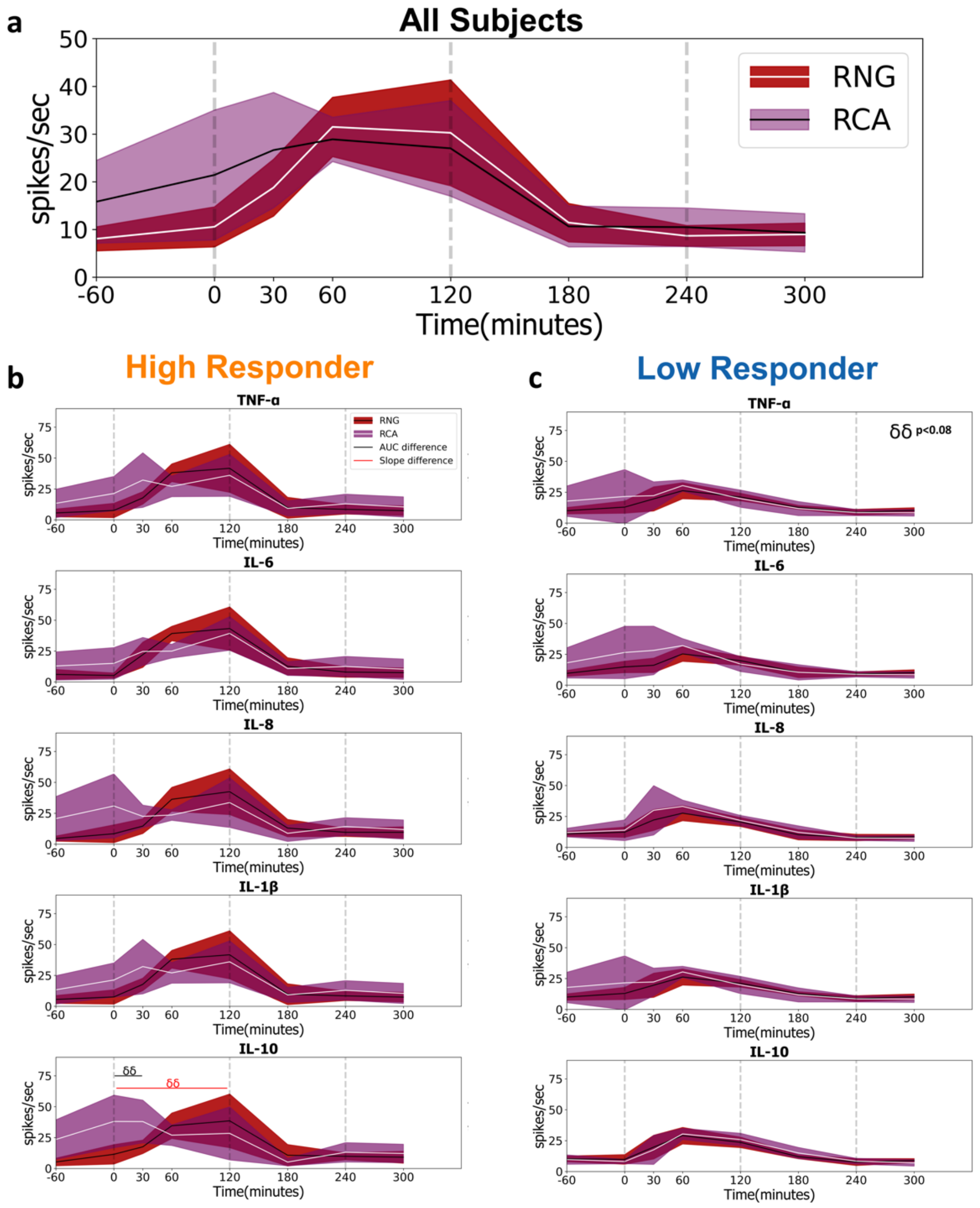

***Supplementary Figure 6**: Change in RNG and RCA MNG activity pre-to-post injection of LPS for all subjects (Panel a) and for the high and low responder subgroups for each cytokine (Panel b and c).* *The shaded areas represented the 95% confidence interval. The area under the curve (AUC) difference for each session between two MNG activities was marked by black lines and black symbols above each session if it attained significance. RNG and RCA MNG activities only showed trend-level differences with respect to AUC during the 0–30-min interval for the IL-10 high responder subgroup. The slope difference during the first period of increase (0-120 minutes) and onset of decrements in the middle period (120-240 minutes) was identified with red lines and red symbols above the corresponding significant period* (δδ =p<.08). *Trend-level differences in slope difference were observed only for the IL-10 high responder subgroup during the initial period with increasing levels.*

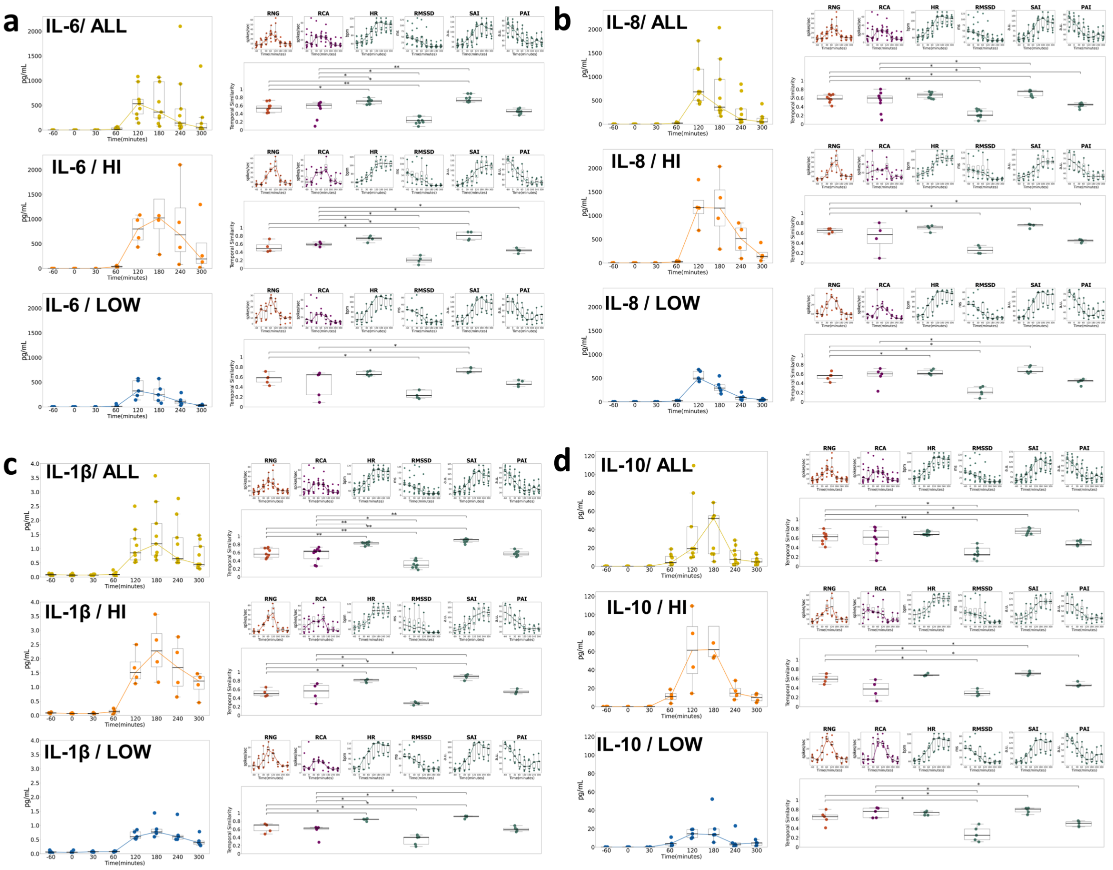

Supplementary Figure 7: Normalized template matching between cytokine concentrations (IL-6, IL-8, IL-1β and IL-10) and RNG, RCA, Heart rate, RMSSD, SAI, and PAI metrics for all subjects (top row of each sub-figure), high responder subgroup (middle row of each sub-figure), and low responder subgroup (bottom row of each sub-figure). *Wilcoxon Rank Sum tests were used to examine RNG, RCA, and each individual cardiac-related metric, and significance was conveyed by connected segments above each box plot pair (* p<0.05, ** P<0.01). The template matching analysis is meant to reflect different physiological signals that covary in time. HR and SAI have the highest similarity with the cytokines in this figure (IL-6, IL-8, IL-1β, IL-10) that reach their maximum peak later in the trial because their own maxima also occur later in time. The higher similarity between the other physiological measurements and the latter cytokines is not an indication of directionality or causality. However, the relationship between TNF-α and RNG is more likely to be physiologically relevant because of the presence of TNF-α receptors on the vagus nerve. Boxplot centerline: median; box limits: Q1 and Q3 quantile.*
