# Supplementary material for: Non-invasive ventral cervical magnetoneurography as a proxy of in vivo lipopolysaccharide-induced inflammation
Source: Commun Biol. 2024 Jul 29;7:893. doi: 10.1038/s42003-024-06435-8 (PMC11286963; doi:10.1038/s42003-024-06435-8)
Supplement: Supplementary file 2 — Description of Additional Supplementary Materials [file 42003_2024_6435_MOESM2_ESM.docx]

**Description of Additional Supplementary Files**

**File name:** Supplementary Data

**Description:** The source data used to generate graphs in the paper
