# Supplementary material for: Non-invasive ventral cervical magnetoneurography as a proxy of in vivo lipopolysaccharide-induced inflammation
Source: Commun Biol. 2024 Jul 29;7:893. doi: 10.1038/s42003-024-06435-8 (PMC11286963; doi:10.1038/s42003-024-06435-8)
Supplement: Supplementary file 4 — Reporting summary [file 42003_2024_6435_MOESM4_ESM.pdf]

nature portfolio

Corresponding author(s): Imaneul Leman

Last updated by author(s): Apr 26, 2024

# Reporting Summary

Nature Portfolio wishes to improve the reproducibility of the work that we publish. This form provides structure for consistency and transparency in reporting. For further information on Nature Portfolio policies, see our Editorial Policies and the Editorial Policy Checklist.

## Statistics

For all statistical analyses, confirm that the following items are present in the figure legend, table legend, main text, or Methods section.

| n/a | Confirmed | |
|---|---|---|
| ☐ | ☒ | The exact sample size (*n*) for each experimental group/condition, given as a discrete number and unit of measurement |
| ☐ | ☒ | A statement on whether measurements were taken from distinct samples or whether the same sample was measured repeatedly |
| ☐ | ☒ | The statistical test(s) used AND whether they are one- or two-sided<br>*Only common tests should be described solely by name; describe more complex techniques in the Methods section.* |
| ☒ | ☐ | A description of all covariates tested |
| ☐ | ☒ | A description of any assumptions or corrections, such as tests of normality and adjustment for multiple comparisons |
| ☐ | ☒ | A full description of the statistical parameters including central tendency (e.g. means) or other basic estimates (e.g. regression coefficient) AND variation (e.g. standard deviation) or associated estimates of uncertainty (e.g. confidence intervals) |
| ☐ | ☒ | For null hypothesis testing, the test statistic (e.g. *F*, *t*, *r*) with confidence intervals, effect sizes, degrees of freedom and *P* value noted<br>*Give P values as exact values whenever suitable.* |
| ☒ | ☐ | For Bayesian analysis, information on the choice of priors and Markov chain Monte Carlo settings |
| ☒ | ☐ | For hierarchical and complex designs, identification of the appropriate level for tests and full reporting of outcomes |
| ☒ | ☐ | Estimates of effect sizes (e.g. Cohen's *d*, Pearson's *r*), indicating how they were calculated |

*Our web collection on statistics for biologists contains articles on many of the points above.*

## Software and code

Policy information about availability of computer code

| | |
|---|---|
| Data collection | The physiological analog signal from the OPM electronic module was sampled by a CED Micro1401 device at 10 kHz and recorded by Signal 8.19a software (Cambridge Electronic Design, Cambridge, UK) |
| Data analysis | No custom code was used to generate or process the data decribed in the manuscript. |

For manuscripts utilizing custom algorithms or software that are central to the research but not yet described in published literature, software must be made available to editors and reviewers. We strongly encourage code deposition in a community repository (e.g. GitHub). See the Nature Portfolio guidelines for submitting code & software for further information.

## Data

Policy information about availability of data

All manuscripts must include a data availability statement. This statement should provide the following information, where applicable:

- Accession codes, unique identifiers, or web links for publicly available datasets
- A description of any restrictions on data availability
- For clinical datasets or third party data, please ensure that the statement adheres to our policy

The source data used to generate graph in the paper can be found in the Supplementary Data File.

## Research involving human participants, their data, or biological material

Policy information about studies with human participants or human data. See also policy information about sex, gender (identity/presentation), and sexual orientation and race, ethnicity and racism.

| | |
|---|---|
| Reporting on sex and gender | Twelve physically healthy male participants were recruited for this study |
| Reporting on race, ethnicity, or other socially relevant groupings | Ethnicity is reported in Supplementary Table 1 |
| Population characteristics | Median age of 20 yr (18 - 30 yr) and median weight of 71.5 kg (52 -118 kg, BMI: 22.86 ± 4.95) (Supplementary Table 1). On the test day, subjects underwent physical history and exam clearance |
| Recruitment | Participants were recruited by flyers posted in the UCSD university campus and surrounding area |
| Ethics oversight | The Institutional Review Board at the University of California, San Diego Health Systems reviewed and approved the experimental protocol (UCSD IRB #191463) |

Note that full information on the approval of the study protocol must also be provided in the manuscript.

# Field-specific reporting

Please select the one below that is the best fit for your research. If you are not sure, read the appropriate sections before making your selection.

☒ Life sciences ☐ Behavioural & social sciences ☐ Ecological, evolutionary & environmental sciences

For a reference copy of the document with all sections, see nature.com/documents/nr-reporting-summary-flat.pdf

# Life sciences study design

All studies must disclose on these points even when the disclosure is negative.

| | |
|---|---|
| Sample size | Sample size is determined following the published work from Kox, Matthijs, et al. "Interplay between the acute inflammatory response and heart rate variability in healthy human volunteers." Shock 36.2 (2011): 115-120, which also recruited 12 subjects to assess cardiac autonomic activity during an LPS intravenous injection challenge. |
| Data exclusions | One subject was excluded due to exhibiting syncopal signs and symptoms upon IV insertion. Two subjects experienced nausea and emesis after which the magnetically shielded room (MSR) was opened for care prior to halting data acquisition of magnetic field signals. |
| Replication | All subjects are administered with the same dosage of LPS. |
| Randomization | No randomization was required because all subjects were administered with the same dosage of LPS |
| Blinding | No Blinding was required because all subjects were administered with the same dosage of LPS. |

# Reporting for specific materials, systems and methods

We require information from authors about some types of materials, experimental systems and methods used in many studies. Here, indicate whether each material, system or method listed is relevant to your study. If you are not sure if a list item applies to your research, read the appropriate section before selecting a response.

## Materials & experimental systems

| n/a | Involved in the study | |
|---|---|---|
| ☒ | ☐ | Antibodies |
| ☒ | ☐ | Eukaryotic cell lines |
| ☒ | ☐ | Palaeontology and archaeology |
| ☒ | ☐ | Animals and other organisms |
| ☒ | ☐ | Clinical data |
| ☒ | ☐ | Dual use research of concern |
| ☒ | ☐ | Plants |

## Methods

| n/a | Involved in the study | |
|---|---|---|
| ☒ | ☐ | ChIP-seq |
| ☒ | ☐ | Flow cytometry |
| ☒ | ☐ | MRI-based neuroimaging |

## Plants

| | |
|---|---|
| Seed stocks | N/A |
| Novel plant genotypes | N/A |
| Authentication | N/A |
